# Effect of sonication on staphylococcal and gram-negative biofilms relevant to prosthetic joint infections: an in vitro study

**DOI:** 10.1007/s42770-026-01986-w

**Published:** 2026-06-01

**Authors:** Natally Dos Santos Silva, Cynthia Regina Pedrosa Soares, Fábio André Brayner dos Santos, Paulo Sérgio Ramos de Araújo

**Affiliations:** 1 Department of Tropical Medicine, Federal University of Pernambuco - UFPE, Hospital das Clínicas, Ground Floor, Hospital das Clínicas, Av. Prof. Moraes Rego, 1235, University City, Recife, 50670-901 Brazil; 2 Department of Parasitology - Aggeu Magalhães Institute - IAM - Fiocruz-PE, Cidade Universitária, CEP, Avenida Professor Moraes Rego, s/n, Recife, 50.740-465 Brazil

**Keywords:** Biofilm, Sonication, Staphylococci, Gram-negative bacteria, Prosthetic Joint Infection

## Abstract

**Background:**

Periprosthetic joint infections (PJIs) are severe complications of arthroplasty in which biofilm formation on implant surfaces compromises microbiological diagnosis and antimicrobial efficacy. Although staphylococci remain the predominant pathogens, Gram-negative bacilli have increasingly been associated with diagnostic failure and unfavorable clinical outcomes. This study aimed to evaluate the formation, maturation, and structural organization of Gram-positive and Gram-negative bacterial biofilms and to investigate the effects of a standardized sonication protocol on biofilm disruption.

**Methods:**

Biofilms of *Staphylococcus aureus* (ATCC 43300), *Staphylococcus epidermidis* (ATCC 35984), *Escherichia coli* (ATCC 25922), and *Pseudomonas aeruginosa* (ATCC 53278) were formed on polyethylene catheter segments for 24, 48, and 72 h and analyzed by scanning electron microscopy (SEM). A standardized sonication protocol was applied to disrupt the extracellular polymeric substance (EPS) matrix, and the resulting sonication fluid was subsequently cultured. Complementary semi-quantitative image-based analysis was performed to compare structural changes after sonication.

**Results:**

All species developed progressively mature biofilms over time, with increased structural complexity and EPS accumulation at later stages. Gram-positive bacteria formed denser and more compact biofilms, whereas Gram-negative species exhibited more heterogeneous and multilayered architectures. Sonication consistently disrupted biofilm integrity across all species and maturation stages, leading to fragmentation of the EPS matrix and increased cellular dispersion. Semi-quantitative image-based analysis suggested greater dispersion in Gram-negative biofilms and reduction of biofilm structural area in Gram-positive species, with more pronounced effects observed in *P. aeruginosa* and *S. epidermidis*. Bacterial growth was observed in cultures obtained from the sonication fluid after the procedure.

**Conclusion:**

The standardized sonication protocol effectively disrupted biofilms formed by both Gram-positive and Gram-negative bacteria, promoting the release of bacterial cells from the biofilm matrix and reinforcing the potential role of sonication as a complementary diagnostic approach for prosthetic joint infections.

**Supplementary Information:**

The online version contains supplementary material available at 10.1007/s42770-026-01986-w.

## Introduction

Periprosthetic joint infections (PJIs) represent one of the most severe complications following arthroplasty, with significant impacts on patient morbidity, mortality, and healthcare costs [1, 2]. Although *Staphylococcus aureus* and coagulase-negative staphylococci remain the predominant etiological agents, Gram-negative bacilli, particularly *Escherichia coli* and *Pseudomonas aeruginosa*, have increasingly been recognized as relevant causes of PJIs and are frequently associated with more aggressive clinical courses and reduced therapeutic responsiveness [3]. In these infections, biofilm formation on implant surfaces plays a central role in persistence, as it protects microorganisms from host immune responses and markedly reduces antimicrobial efficacy.

Biofilm development is a dynamic and structured process that includes initial adhesion, maturation with extracellular polymeric substance (EPS) production, and dispersion [4–6]. As biofilms mature, antimicrobial tolerance increases and the sensitivity of conventional diagnostic methods declines, leading to frequent diagnostic failures, particularly in chronic infections assessed solely by periprosthetic tissue culture (PTC), which remains the diagnostic gold standard in many settings [5–7].

To address these limitations, physical methods for biofilm disruption, particularly sonication, have been incorporated into diagnostic workflows. Since its initial description by Trampuz et al. in 2007, sonication has demonstrated improved diagnostic performance compared with PTC in both experimental and clinical studies [8, 9]. However, considerable heterogeneity in sonication protocols has resulted in inconsistent evidence regarding its diagnostic benefit [8, 10, 11]. Moreover, few studies have provided direct SEM-based visualization of the effects of sonication on biofilms formed by Gram-negative bacteria at different stages of maturation.

Previously, we proposed a standardized sonication protocol based on a comprehensive literature analysis [11], but its experimental application had not been evaluated. Therefore, the present study aimed to investigate, in vitro, the effects of sonication on biofilms formed by *Staphylococcus aureus*, *Staphylococcus epidermidis*, *Escherichia coli*, and *Pseudomonas aeruginosa* at different maturation stages using scanning electron microscopy, within a model relevant to PJIs.

## Materials and methods

Laboratory reference strains of *S. epidermidis* (ATCC 35984), *S. aureus* (ATCC 43300), *E. coli* (ATCC 25922), and *P. aeruginosa* (ATCC 53278) were used to ensure experimental reproducibility and minimize phenotypic variability in biofilm formation. The strains were pre-cultured in 2 to 5 mL of Brain Heart Infusion (BHI) broth at 37 °C for 18–24 h, and the inocula were standardized to approximately 1 × 10⁸ CFU/mL.

Biofilms were formed on sterile polyethylene catheter segments (~ 1.5 cm in length) obtained from the distal (thinner) portion of a conventional central venous catheter (approximately 2.0 mm outer diameter), incubated statically in 2 mL of BHI at 37 °C for 24, 48, or 72 h, corresponding to early, intermediate, and mature stages of biofilm development. After 24 h, samples were washed with sterile saline to remove planktonic cells, transferred to fresh medium, and incubated under the same conditions, with daily medium renewal. For each strain and time point, sonicated and non-sonicated biofilms were analyzed, along with sterile negative controls. All experiments were performed in triplicate.

Sonication was performed according to a previously standardized protocol [11]. Briefly, catheter segments were vortexed for 30 s, sonicated in an ultrasonic bath for 1 min at 40 ± 2 kHz (0.22 ± 0.04 W/cm²), and vortexed again for 30 s. Sonication fluid was centrifuged (3200 rpm, 15 min), and a 10 µL aliquot of sonication was cultured on BHI agar to assess bacterial growth after sonication.

For scanning electron microscopy (SEM), catheter segments were fixed in 2.5% glutaraldehyde, post-fixed in 1% osmium tetroxide, dehydrated in graded ethanol, sputter-coated with gold, and imaged to assess biofilm architecture and the effects of sonication. Multiple fields were examined for each condition.

The impact of sonication on bacterial biofilms was evaluated through qualitative and quantitative analyses based on scanning electron microscopy (SEM) images. The qualitative analysis consisted of visual comparison between control and sonicated biofilms, aiming to characterize biofilm architecture, structural integrity, and morphological changes associated with sonication.

The semi-quantitative analysis was performed using ImageJ^®^ software (National Institutes of Health, USA), which enables measurement of areas/perimeters and cellular quantification in scientific images. For Gram-positive bacteria (*S. aureus* and *S. epidermidis*), quantification was based on the mean area of biofilm structures. For Gram-negative bacteria (*E. coli* and *P. aeruginosa*), which exhibited greater cellular dispersion after sonication, quantification was based on cell density, calculated as the number of cells per total analyzed field area (cells/µm²).

All analyses were performed in triplicate, using three independent SEM images per condition (with and without sonication) for each species, and the data were organized in a table (Table S1) and presented as bar graphs (mean ± standard deviation) using GraphPad Prism^®^ (GraphPad Software, USA), allowing comparison across species and experimental conditions (with and without sonication).

Additionally, an exploratory sonication impact index was calculated as the ratio between values observed in sonicated and control groups, providing a comparative semi-quantitative assessment of the relative structural effects of sonication on the analyzed biofilms. For Gram-positive bacteria, the index was derived from the ratio between the mean area of biofilm structures in sonicated and control groups. For Gram-negative bacteria, the index was calculated based on the ratio between cell densities observed after sonication and their respective controls. Values greater than 1 suggested increased dispersion or fragmentation associated with sonication, whereas values closer to 1 suggested a lower relative structural effect.

## Results

**Fig. 1:**
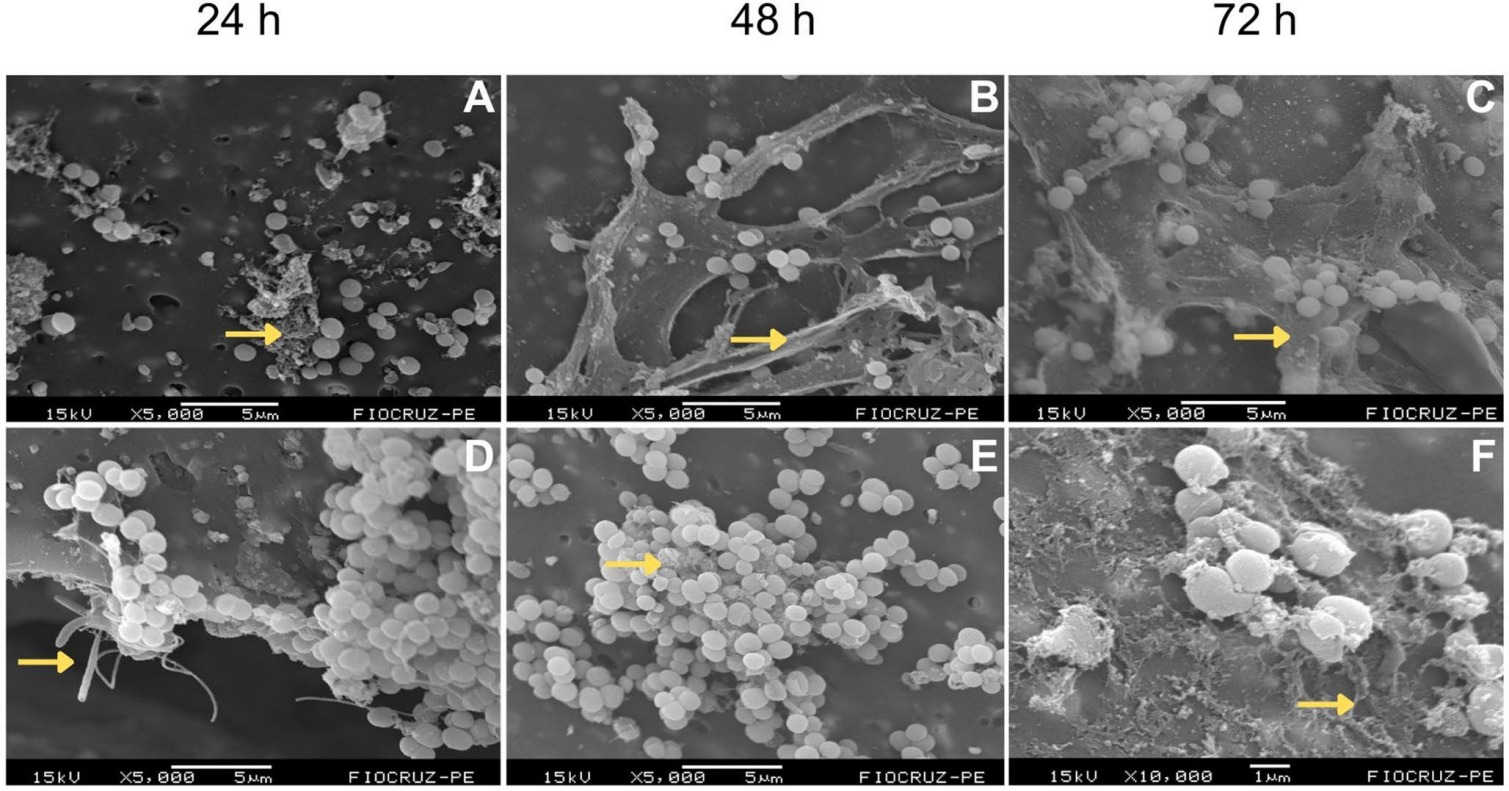
Temporal evolution of biofilm formation by *Staphylococcus aureus* (**A**–**C**) and *Staphylococcus epidermidis* (**D**–**F**) at 24, 48 and 72 h. Arrows indicate regions of EPS matrix associated with bacterial cells. Scale bars are shown within each panel

**Fig. 2:**
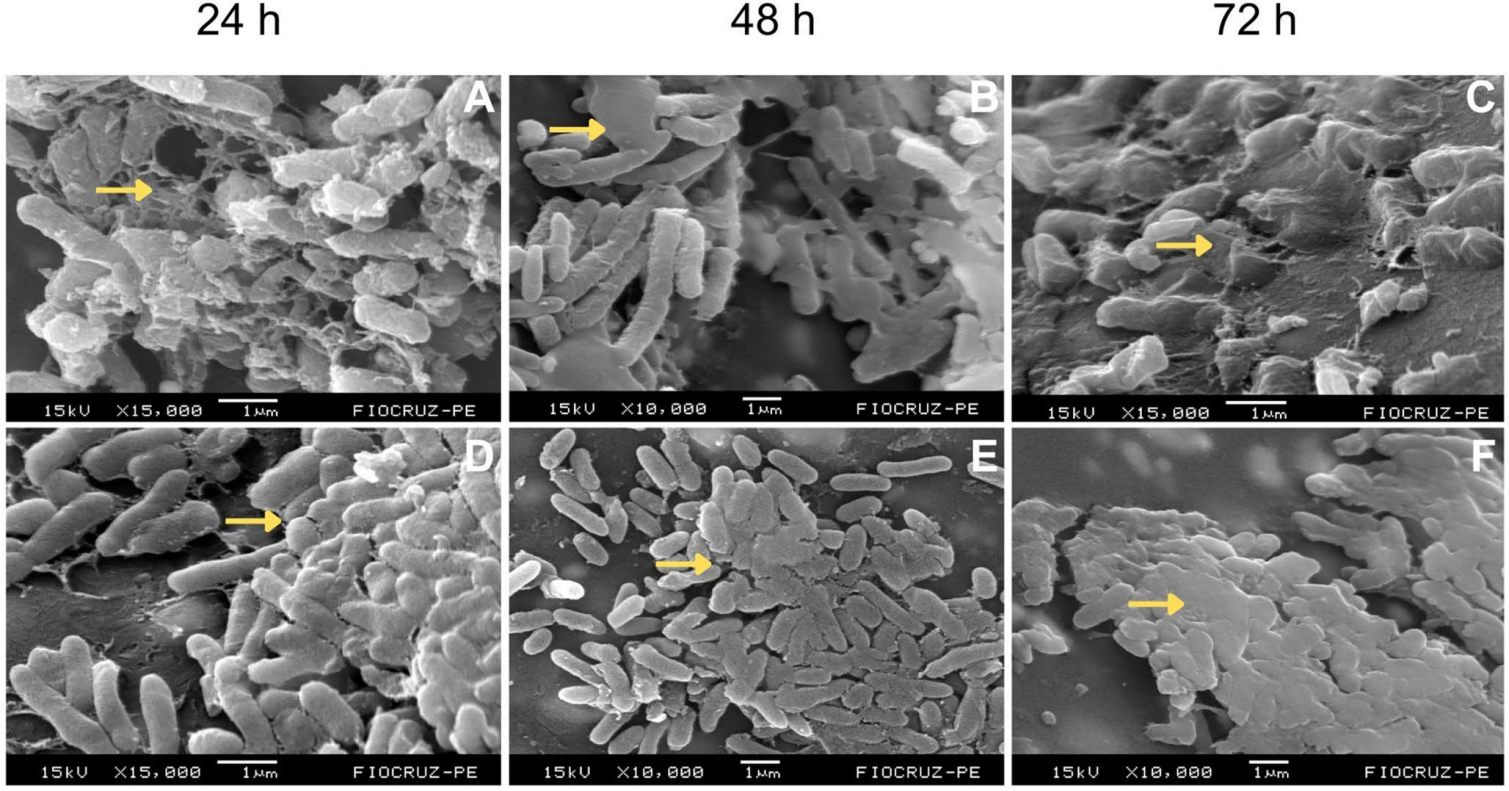
Temporal evolution of biofilm formation by *Escherichia coli* (**A**–**C**) and *Pseudomonas aeruginosa* (**D**–**F**) at 24, 48 and 72 h. Arrows indicate regions of EPS matrix associated with bacterial cells. Scale bars are shown within each panel

All strains tested in this study, *S. epidermidis* (ATCC 35984), *S. aureus* (ATCC 43300), *E. coli* (ATCC 25922) and *P. aeruginosa* (ATCC 53278), successfully formed biofilms following the proposed methodology, exhibiting characteristic structural organization. The biofilms consisted of dense, highly hydrated aggregates separated by interstitial spaces or water channels, forming a three-dimensional (3D) architecture embedded in an extracellular polymeric substance that appears as an amorphous material under scanning electron microscopy (SEM). Across all incubation periods evaluated (24, 48, and 72 h), each strain exhibited distinct stages of biofilm maturation, with progressive increases in density, complexity, and three-dimensional organization over time (Figs. 1 and 2).

The biofilm formed by *S. aureus* exhibited, after 24 h, sparse coccoid cells arranged in small aggregates of bacterial cells and EPS matrix, interspersed with areas of exposed surface and surrounded by an incipient amorphous material, consistent with initial adhesion and microcolony formation (Fig. 1A). After 48 h, the biofilm architecture became more complex, with larger clusters of bacterial cells interconnected by abundant EPS, forming filamentous and web-like structures that bridged adjacent microcolonies (Fig. 1B). After 72 h, a mature biofilm was observed, characterized by densely packed cellular aggregates partially embedded within a thick and continuous extracellular matrix, conferring a well-defined three-dimensional organization to the biofilm (Fig. 1C).

Similarly, *S. epidermidis* demonstrated early adhesion at 24 h, with coccoid cells forming compact clusters and prominent filamentous extracellular structures extending from the biofilm surface, indicating active matrix production during the initial stages of biofilm development (Fig. 1D). At 48 h, the biofilm exhibited a marked increase in cellular density, with densely packed bacterial aggregates embedded within a more homogeneous and cohesive extracellular matrix (Fig. 1E). At 72 h, despite a lower number of cells, a mature biofilm was evident, characterized by bacterial clusters enveloped by an abundant extracellular matrix, forming a robust and stable architecture that covered most of the analyzed surface (Fig. 1F).

Among the Gram-negative bacteria analyzed, *E. coli* exhibited, at 24 h, a structure composed of well-individualized and dispersed cells surrounded by a thin amorphous layer, consistent with the adhesion phase and the onset of microcolony formation. At 48 h and 72 h, the biofilms displayed more aggregated bacterial cells embedded within an increasingly dense amorphous matrix. By 72 h, many cells were partially submerged within this structure, which is a hallmark of a mature biofilm (Fig. 2A–C).

The 24-h biofilm of *P. aeruginosa* showed early adhesion of irregularly arranged rod-shaped cells and the initial accumulation of visible EPS. At 48 h, the biofilm exhibited larger and denser bacterial clusters with a heterogeneous organization, characterized by small three-dimensional elevations separated by uncovered areas. By 72 h, a mature biofilm was evident, characterized by a compact cellular mass covered by a thick, continuous EPS matrix extending across nearly the entire surface (Fig. 2D–F).

Overall, *P. aeruginosa* biofilms were consistently thicker and exhibited greater structural complexity across all analyzed time points when compared to those formed by the other strains.

Regarding the effect of the sonication protocol on biofilm structure, we observed that sonication consistently disrupted the extracellular matrix in all strains and maturation stages evaluated, promoting biofilm disaggregation and the release of bacterial cells from the biofilm structure.

**Fig. 3:**
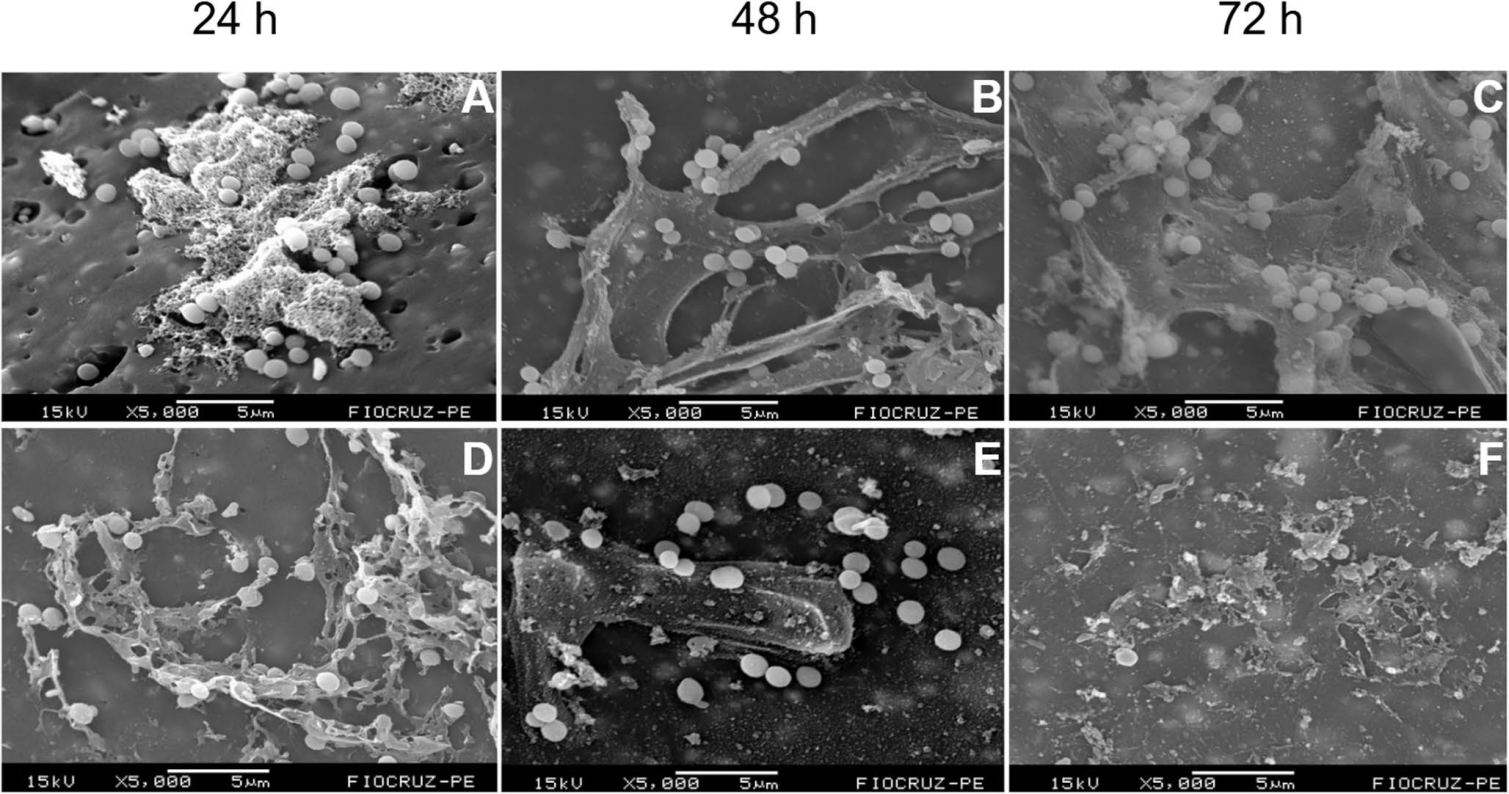
Structural effect of the sonication protocol on *Staphylococcus aureus* biofilms at 24, 48 and 72 h. Panels A–C show non-sonicated biofilms at 24, 48 and 72 h, respectively, while panels D–F depict the corresponding sonicated samples, illustrating matrix disruption, reduced aggregation, and increased dispersion of bacterial cells. Scale bars are shown within each panel

In *S. aureus* biofilms, sonication promoted substantial structural disruption at all stages of biofilm maturation, although complete removal of the biofilm was not observed (Fig. 3). In the 24-h biofilm, sonication led to the disorganization of early aggregates and partial detachment of coccoid cells, with fragmentation of the incipient extracellular matrix and increased dispersion of individual cells across the surface (Fig. 3D). In the 48-h biofilm, which exhibited a highly organized filamentous matrix in the control samples, sonication caused pronounced rupture of the extracellular network, resulting in discontinuous matrix remnants, loss of intercellular connections, and reduced cohesion of bacterial clusters (Fig. 3E). In the mature 72-h biofilm, sonication induced marked architectural damage, characterized by extensive matrix disruption, breakdown of dense cellular aggregates, and the presence of scattered coccoid cells still partially associated with residual fragments of the extracellular matrix (Fig. 3F).

**Fig. 4:**
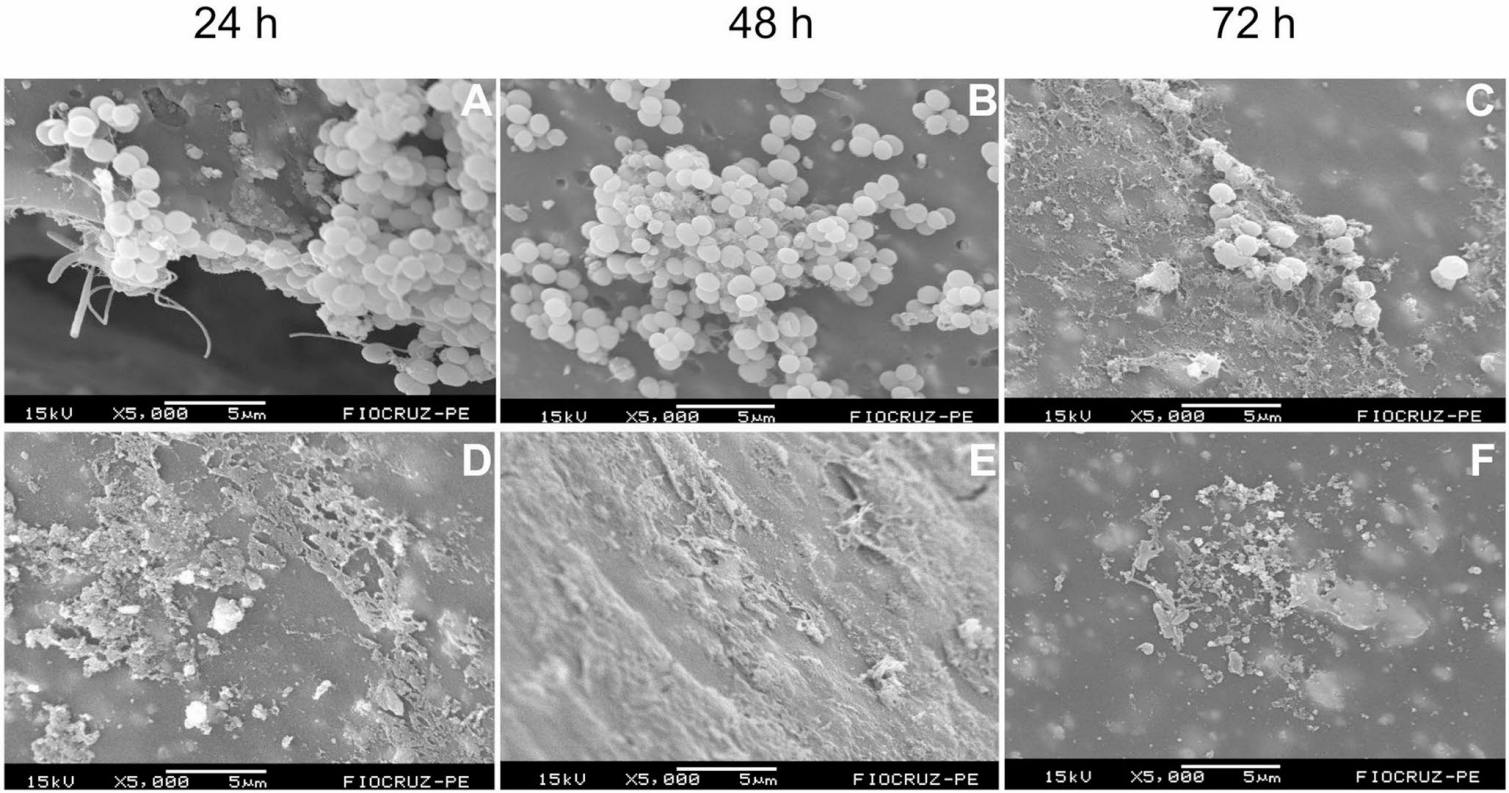
Structural effect of the sonication protocol on *Staphylococcus epidermidis* biofilms at 24, 48 and 72 h. Panels A–C show non-sonicated biofilms at 24, 48 and 72 h, respectively, while panels D–F depict the corresponding sonicated samples, illustrating matrix disruption, reduced aggregation, and increased dispersion of bacterial cells. Scale bars are shown within each panel

For *S. epidermidis*, the effects of sonication were also evident but differed in magnitude and pattern when compared to *S. aureus* (Fig. 4). At 24 h, sonication disrupted the compact cellular clusters observed in the control biofilms, leading to partial detachment and fragmentation of the filamentous extracellular structures, although portions of the matrix remained adhered to the surface (Fig. 4D). In the 48-h biofilm, sonication resulted in extensive surface clearing, with a marked reduction in adherent bacterial aggregates and the predominance of isolated coccoid cells and amorphous matrix residues (Fig. 4E). In the mature 72-h biofilm, despite the pronounced disruption of the biofilm architecture, residual cellular aggregates and extracellular matrix fragments persisted, indicating a high degree of structural resilience of S. epidermidis biofilms to the sonication protocol (Fig. 4F).

**Fig. 5:**
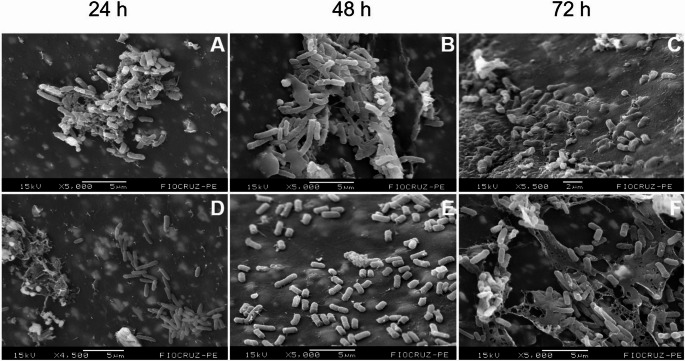
Structural effect of the sonication protocol on *Escherichia coli* biofilms at 24, 48 and 72 h. Panels (**A**–**C**) show non-sonicated biofilms at 24, 48 and 72 h, respectively, while panels (**D**–**F**) depict the corresponding sonicated samples, illustrating matrix disruption, reduced aggregation, and increased dispersion of bacterial cells. Scale bars are shown within each panel

In *E. coli* biofilms, sonication effectively disrupted the biofilm architecture at all three maturation stages, eliminating the organized structures observed in the corresponding controls (Fig. 5). In the 24-h biofilm, sonication resulted in a clear loss of initial organization, with increased cell dispersion and the absence of compact aggregates. In the 48-h biofilm, this disruptive effect persisted, leading to fragmentation of the developing biofilm and a marked reduction in adherent cellular layers. In the mature 72-h biofilm, an even greater degree of structural damage was observed, consistent with the higher complexity of the three-dimensional architecture in the control samples. This stage was characterized by extensive extracellular matrix disruption, fragmented areas, residual filamentous structures, and dispersed rod-shaped cells, some of which remained attached to fragments of the ruptured matrix (Fig. 5F).

**Fig. 6:**
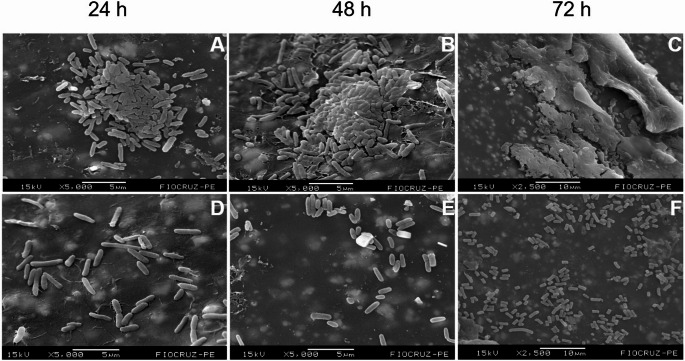
Structural effect of the sonication protocol on *Pseudomonas aeruginosa* biofilms at 24, 48 and 72 h. Panels (**A**–**C**) show non-sonicated biofilms at 24, 48 and 72 h, respectively, while panels (**D**–**F**) depict the corresponding sonicated samples, illustrating matrix disruption, reduced aggregation, and increased dispersion of bacterial cells. Scale bars are shown within each panel

For *P. aeruginosa* biofilms, the effects of the sonication protocol were similarly evident at all maturation stages (Fig. 6). In well-structured biofilms, such as those formed at 48 h, sonication led to extensive fragmentation, with a predominance of individual bacterial cells and an almost complete absence of visible extracellular matrix (Fig. 6E). A comparable pattern was observed in the 72-h biofilm: while the control samples exhibited a dense and mature biofilm architecture, the sonicated samples showed dispersed cellular aggregates and only small residual fragments of the matrix (Fig. 6F).

As demonstrated in the results described above, sonication promoted the release of microbial cells from the biofilm into the sonication fluid, further evidenced by the formation of a visible pellet after centrifugation of the catheter segments together with the rinsing fluid used in the protocol (Fig S27A). The fluid from all samples was subsequently cultured on BHI agar plates, and after 18–24 h of incubation at 37 °C, well-defined bacterial colonies were observed (Fig S27B), confirming that the released cells remained culturable following the procedure.

**Fig. 7:**
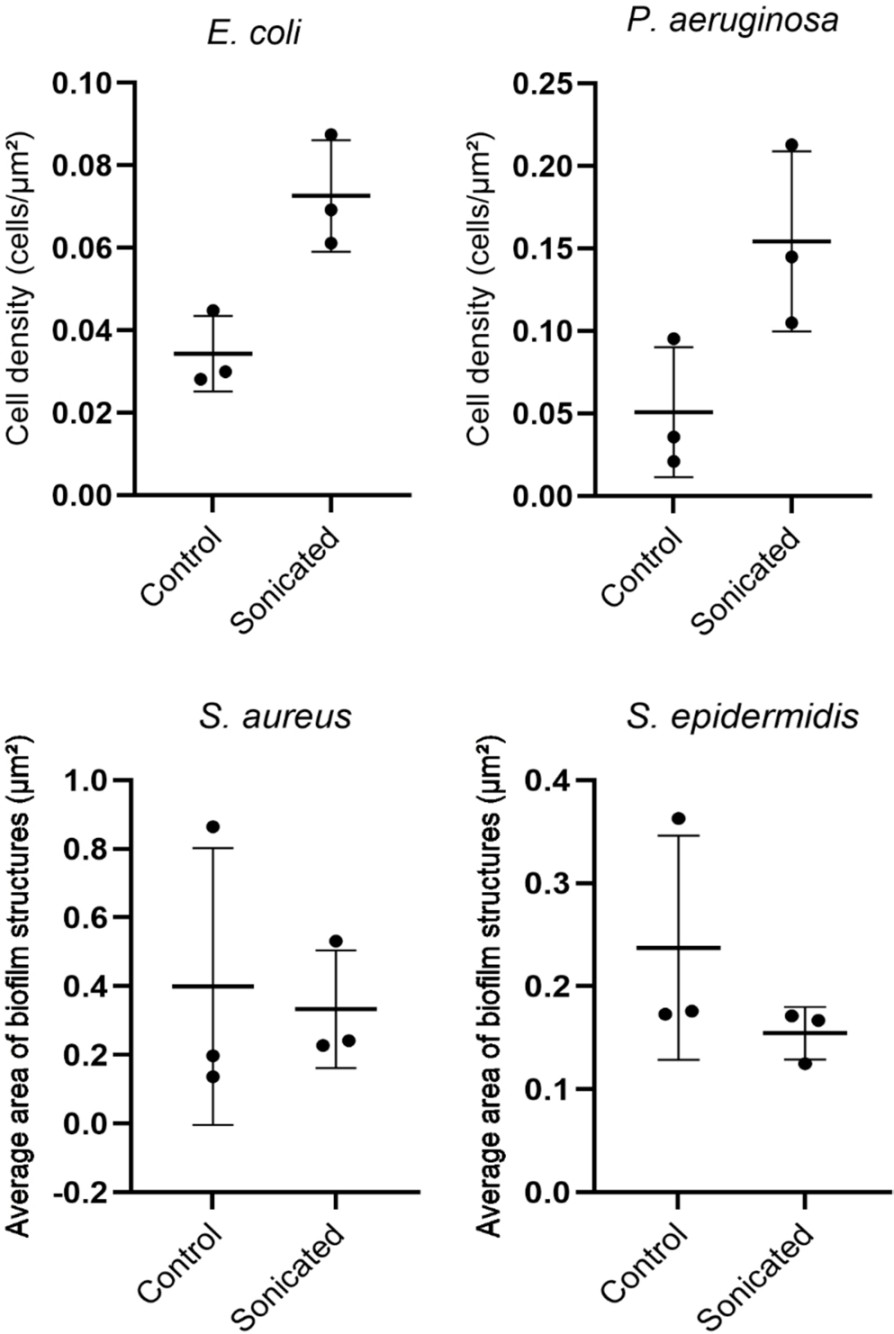
Semi-quantitative analysis of the relative impact of sonication on biofilms of the tested bacterial species. Note: Cell density was used for Gram-negative species, and mean biofilm structural area for Gram-positive species. Data are presented as individual values and mean ± standard deviation

The semi-quantitative analysis of the impact of sonication (Fig. 7) suggested distinct patterns among biofilms formed by Gram-negative and Gram-positive bacteria. In *E. coli*, a consistent increase in cell density was observed after sonication compared to the control, indicating biofilm dispersion and release of previously embedded cells. A similar pattern was observed in *P. aeruginosa*, which showed a marked increase in cell density after sonication, although with greater variability among images, reflecting heterogeneity in biofilm disaggregation.

In Gram-positive bacteria, the impact on biofilm structure was less pronounced but still associated with a reduction in the mean area of biofilm structures. In *S. aureus*, structural area decreased after sonication, accompanied by high variability in the control group, suggesting the presence of more robust and heterogeneous biofilms prior to treatment. In *S. epidermidis*, the reduction in mean area was more uniform, indicating greater susceptibility of the extracellular matrix to mechanical disruption.

The exploratory sonication impact index supported these observations. Among Gram-negative bacteria, the effect was greater in *P. aeruginosa* (index = 3.04) than in *E. coli* (index = 2.12), indicating higher cellular dispersion. Gram-positive species showed lower index values, with *S. epidermidis* (1.54) being more affected than *S. aureus* (1.20).

## Discussion

Biofilms are complex three-dimensional structures formed when microorganisms irreversibly adhere to a surface and produce an extracellular polymeric matrix composed of polysaccharides, proteins, lipids, DNA, and RNA [12, 13]. Biofilm formation, particularly by the species analyzed in this study, plays a central role in the pathogenesis of prosthetic joint infections (PJIs), as it enables evasion of host immune responses, limits the effectiveness of antimicrobial therapy, and reduces the sensitivity of culture-based diagnostic methods [13, 14].

In the present in vitro model, Gram-negative bacteria formed more extensive and volumetric biofilms than Gram-positive bacteria. This difference likely reflects distinct biofilm development strategies, with Gram-negative species exhibiting rapid surface colonization and expansive extracellular matrix production, while staphylococcal biofilms were characterized by compact cell aggregation and a highly cohesive matrix, resulting in reduced lateral expansion.

Among Gram-negative bacteria, *P. aeruginosa* developed robust biofilm structures (Fig. S7–S11), consistent with previous studies demonstrating its high growth and biofilm-forming capacity both in vitro and in vivo [15, 16]. This species forms exceptionally complex and resilient biofilms due to a combination of structural and regulatory features, including an extracellular matrix composed of three major exopolysaccharides—alginate, Psl, and Pel—which act synergistically to generate a dense, cohesive, and highly stable architecture. In addition, gene regulation via cyclic di-GMP and quorum sensing coordinates the expression of genes involved in increased density, maturation, and three-dimensional organization [12].

*E. coli* also efficiently formed biofilms with a characteristic architecture (Fig. S1–S5), although these structures were less organized and exhibited lower complexity than those produced by *P. aeruginosa* [4, 12]. A similar trend was observed for staphylococcal biofilms (Fig. S13–S26), which is consistent with previous in vitro studies showing that *P. aeruginosa* often produces greater biofilm biomass than *S. aureus* under comparable laboratory conditions and may even inhibit staphylococcal biofilm formation in co-culture models [17].

Although this study used polyethylene catheter segments, a material also employed as an articulating surface in combination with metallic or ceramic components in orthopedic prostheses, the species evaluated here, such as *P. aeruginosa* and *S. aureus*, have been shown to form biofilms on a wide range of prosthetic materials (e.g., cobalt–chromium, oxinium, and ceramic femoral heads). This indicates that biofilm formation is not restricted to a single surface and is highly relevant in the clinical setting [18].

Qualitative SEM observations, supported by complementary semi-quantitative image-based analysis, showed that biofilms formed by all tested species exhibited visible disruption of their extracellular matrix following sonication, including the structurally resilient biofilms produced by staphylococci. The semi-quantitative findings supported the morphological observations and allowed a comparative assessment of interspecies differences. These results highlight the mechanical effectiveness of sonication in dislodging biofilm-associated bacterial communities from the underlying surface. Similar findings have been previously reported, showing that sonication can outperform chemical methods in removing biofilms formed by *S. aureus*, *S. epidermidis*, *E. coli*, and *P. aeruginosa* from artificial surfaces [7].

As biofilms mature, they exhibit increased structural organization and matrix cohesion [12], which contributes to the greater complexity and tolerance observed in chronic PJIs and makes both diagnosis and treatment more challenging [16, 19–22]. This is particularly relevant for Gram-negative bacteria, such as *P. aeruginosa* and *E. coli*, which are increasingly associated with prosthetic infections and are linked to higher rates of treatment failure, often requiring multiple surgical interventions and prolonged hospitalization, especially when diagnosis is delayed [16, 19, 20].

In our study, sonication demonstrated its ability to disrupt both early biofilms at 24 h (Figures S2, S8, S14, and S21) and mature biofilms at 72 h (Figures S6, S12, S18, and S26) across all tested species. This finding may explain the widely reported ability of sonication to enhance the sensitivity and specificity of prosthesis-associated infection diagnosis, particularly in chronic or low-grade infections with negative or inconclusive culture results [8, 23–26]. It is also important to highlight that one of the main concerns regarding the use of sonication for biofilm removal from explanted implants is the potential loss of microbial culturability following the procedure [14, 27, 28]. In this study, bacterial growth on agar plates from the sonication fluid demonstrated that the method successfully released microorganisms previously adhered to the biofilm matrix, which remained culturable after the procedure. This indicates that their growth capacity was preserved and underscores the adhesive and structured nature of the evaluated species. These findings contribute to the literature by demonstrating that sonication can enhance microbiological detection of implant-associated bacteria, particularly in cases where conventional methods exhibit low sensitivity [9, 11, 14, 24, 29, 30, 31].

This study presents limitations, many of which are inherent to the experimental model. The use of an in vitro system with laboratory reference strains and polyethylene segments, although allowing controlled biofilm formation, experimental reproducibility, and direct visualization of biofilm architecture, does not fully replicate the biomechanical and immunological complexity of the in vivo microenvironment of infected prostheses, which may influence biofilm organization, maturation, and tolerance. Furthermore, although polyethylene enabled standardized biofilm formation and reproducible SEM analysis, it does not fully represent the physicochemical properties of commonly used orthopedic implant materials, such as titanium or cobalt–chromium alloys. Therefore, the translational applicability of these findings should be interpreted with caution.

The use of reference strains rather than clinical isolates from PJIs represents an additional limitation, as biofilm-forming capacity may vary among clinical strains. However, these standardized strains were deliberately selected to ensure experimental reproducibility and to allow controlled assessment of biofilm maturation and the effects of a standardized sonication protocol. Additionally, bacterial recovery after sonication was limited to culturability, assessed by growth on solid media, and did not include metabolic viability assays capable of detecting viable but non-culturable (VBNC) cells that may persist within biofilms.

Although complementary semi-quantitative image-based analysis was included, incorporating measurements of biofilm structural area and cell density, as well as an exploratory sonication impact index, CFU-based quantification of bacteria released after sonication was not performed. Furthermore, SEM-based image analysis does not provide true volumetric assessment of biofilm biomass and should therefore be interpreted as a complementary morphometric approach. These limitations should be addressed in future studies through the incorporation of additional quantitative methodologies, such as CFU counting, confocal laser scanning microscopy (CLSM), and crystal violet staining assays, to enable more comprehensive comparisons of biofilm disruption and bacterial recovery across species.

Nevertheless, these limitations do not compromise the main finding of this study: sonication effectively disrupts both immature and mature biofilms formed by the species most associated with prosthetic joint infections, promoting disruption of their three-dimensional biofilm matrix and bacterial cell detachment. These findings reinforce the potential of sonication as a complementary tool in the microbiological diagnosis of PJIs, particularly in challenging scenarios such as chronic or low-grade infections.

## Conclusions

The standardized sonication protocol demonstrated effectiveness in disrupting the three-dimensional architecture of biofilms formed by both Gram-negative and Gram-positive bacteria associated with PJIs, including *S. aureus*, *S. epidermidis*, *E. coli*, and *P. aeruginosa*, as well as in releasing cultivable bacterial cells across different stages of biofilm maturation. These findings reinforce the role of sonication as a valuable complementary diagnostic approach for prosthesis-associated infections caused by a broad range of clinically relevant pathogens, particularly in chronic or low-grade infections in which conventional culture methods often show reduced sensitivity.

However, although the present results highlight the translational potential of standardized laboratory sonication protocols to improve clinical diagnosis, they should be interpreted in light of the limitations inherent to this study. Future investigations incorporating clinically relevant prosthetic materials, clinical isolates, and quantitative outcome measures are warranted to further validate and optimize the application of sonication protocols in routine clinical practice.

## Supplementary Information

Below is the link to the electronic supplementary material.

Supplementary Material 1 (DOCX 7.79 MB)

## Data Availability

The datasets generated and/or analyzed during the current study are available from the corresponding author on reasonable request.
