# Supplementary material for: Effect of sonication on staphylococcal and gram-negative biofilms relevant to prosthetic joint infections: an in vitro study
Source: Braz J Microbiol. 2026 Jun 1;57(1):161. doi: 10.1007/s42770-026-01986-w (PMC13226771; doi:10.1007/s42770-026-01986-w)
Supplement: Supplementary file 1 — Supplementary Material 1 (DOCX 7.79 MB) [file 42770_2026_1986_MOESM1_ESM.docx]

**Supplementary Materials:**


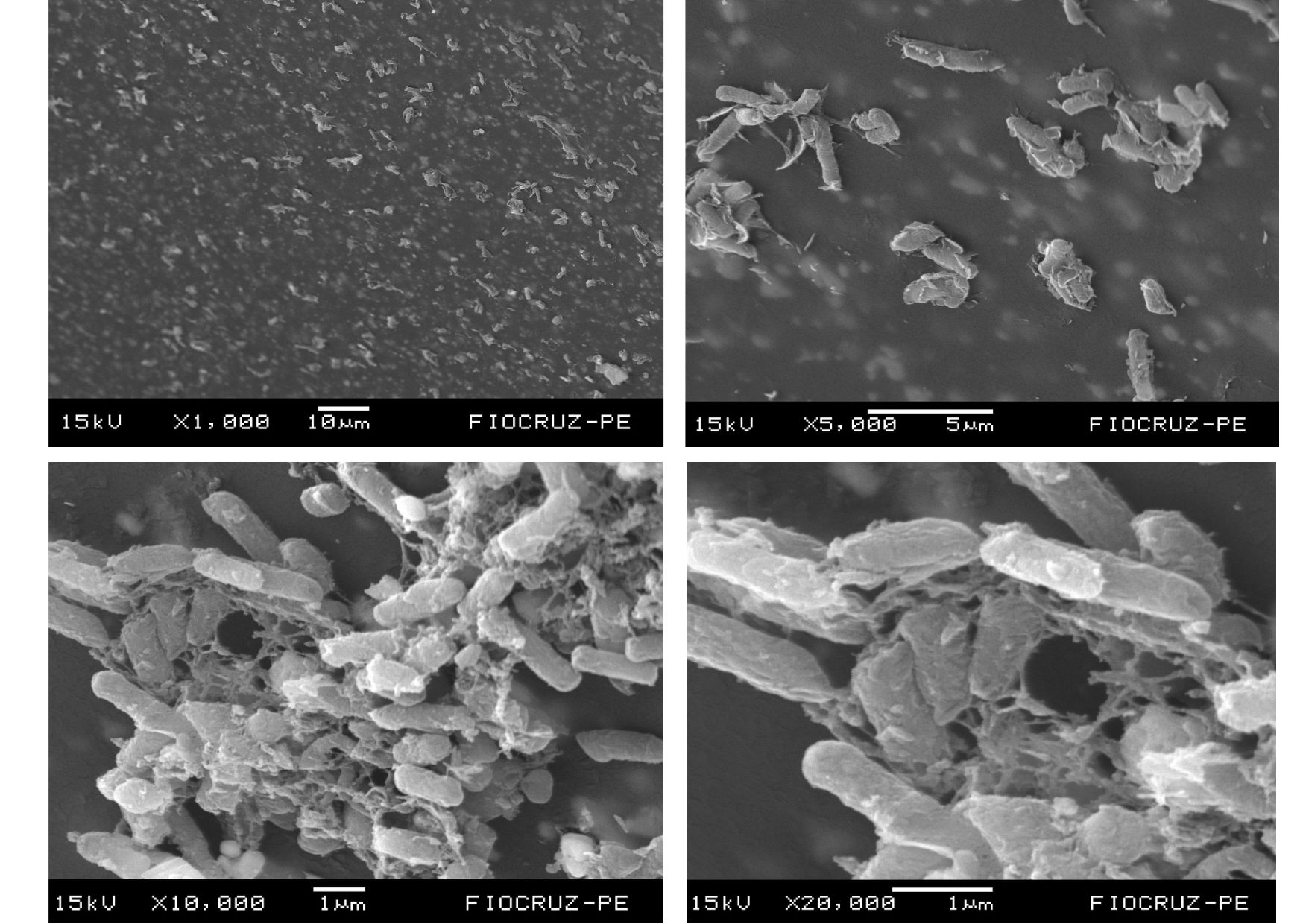


Fig S1. Representative SEM micrographs of 24 h control *Escherichia coli* biofilms at different magnifications (1 000 x, 5 000 x, 10 000 x, 20 000 x).


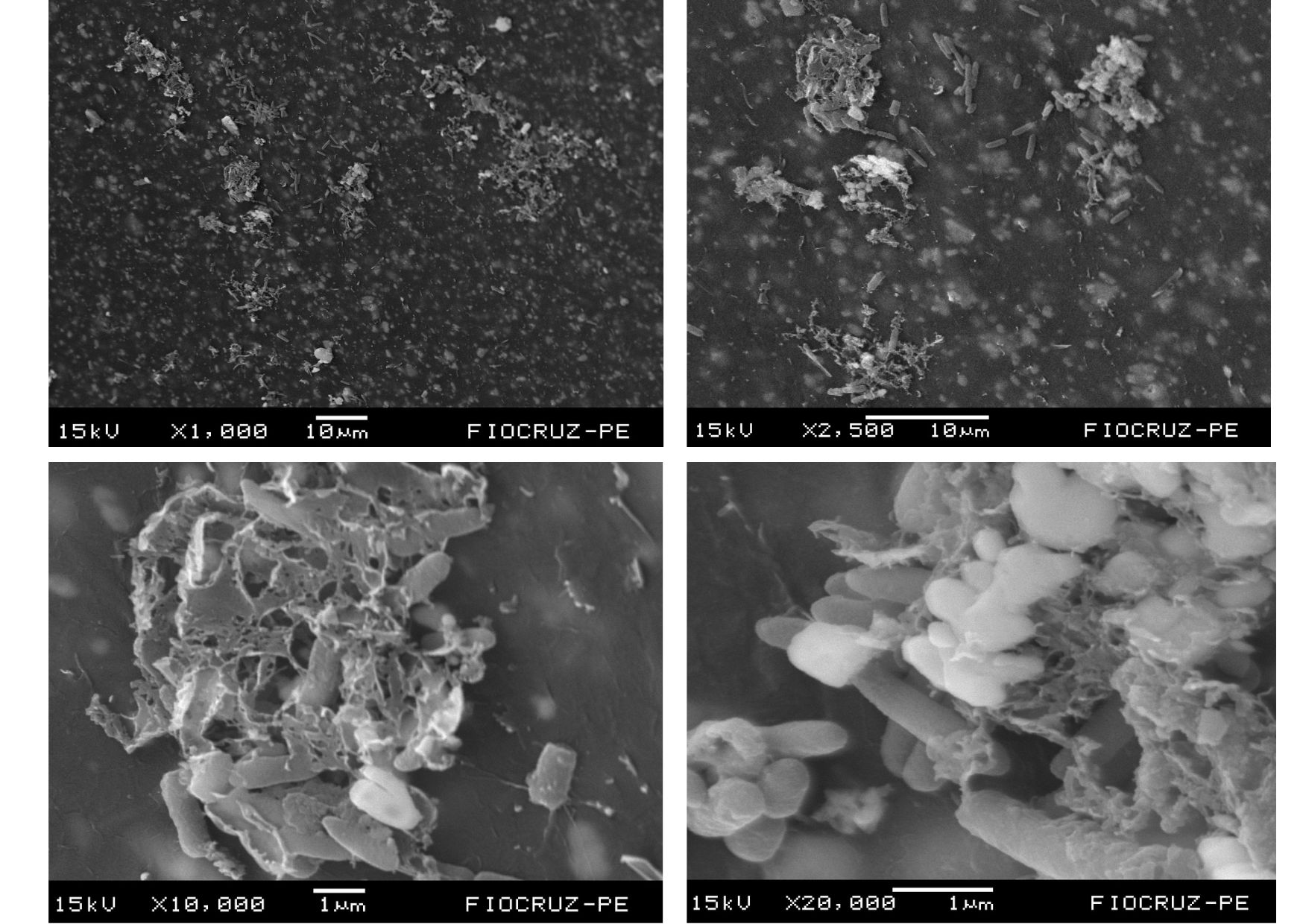


Fig S2. Representative SEM micrographs of 24 h *Escherichia coli* biofilms after sonication treatment at different magnifications (1 000 x, 2 500 x, 10 000 x, 20 000 x).


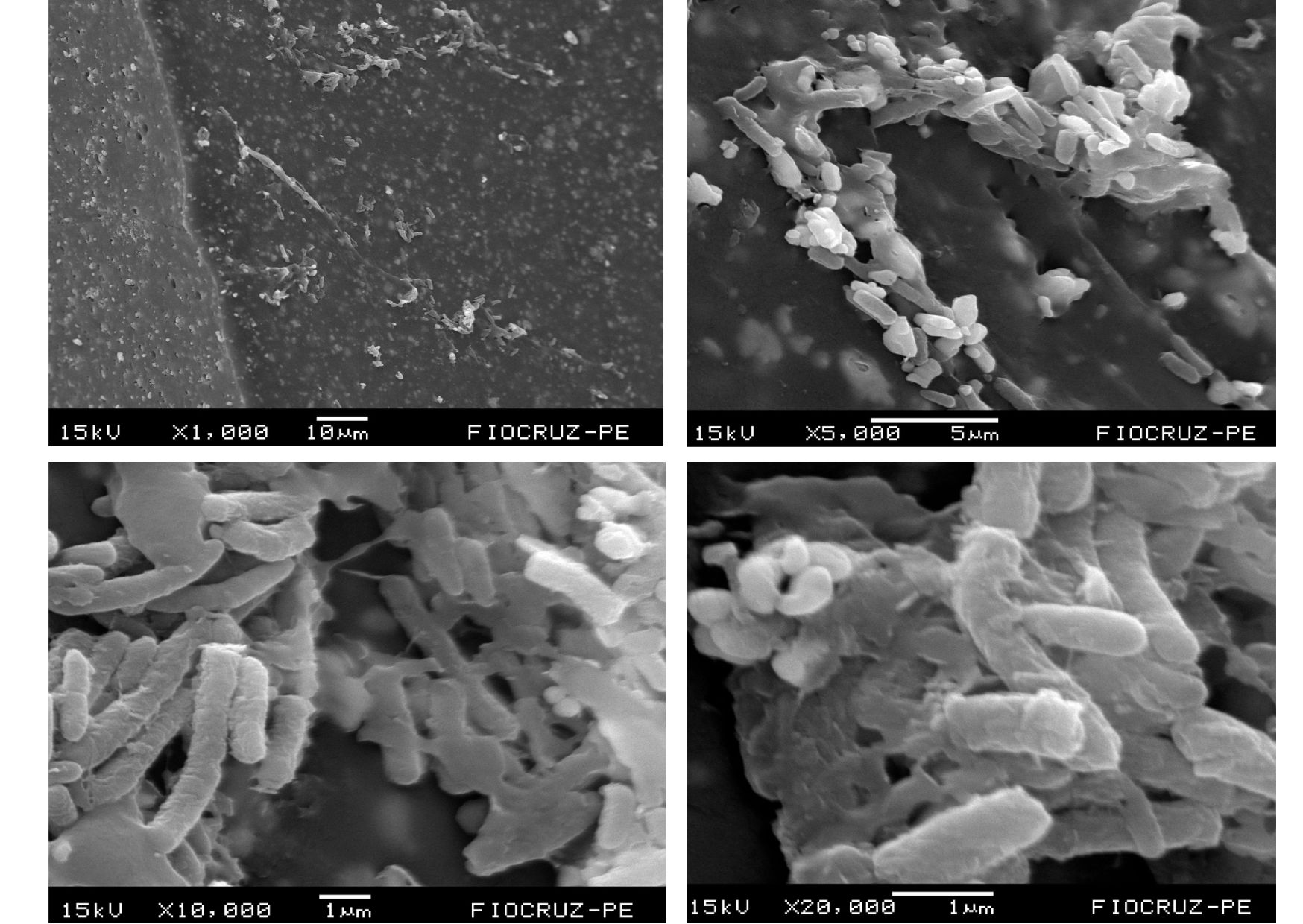


Fig S3. Representative SEM micrographs of 48 h control *Escherichia coli* biofilms at different magnifications (1 000 x, 5 000 x, 10 000 x, 20 000 x).


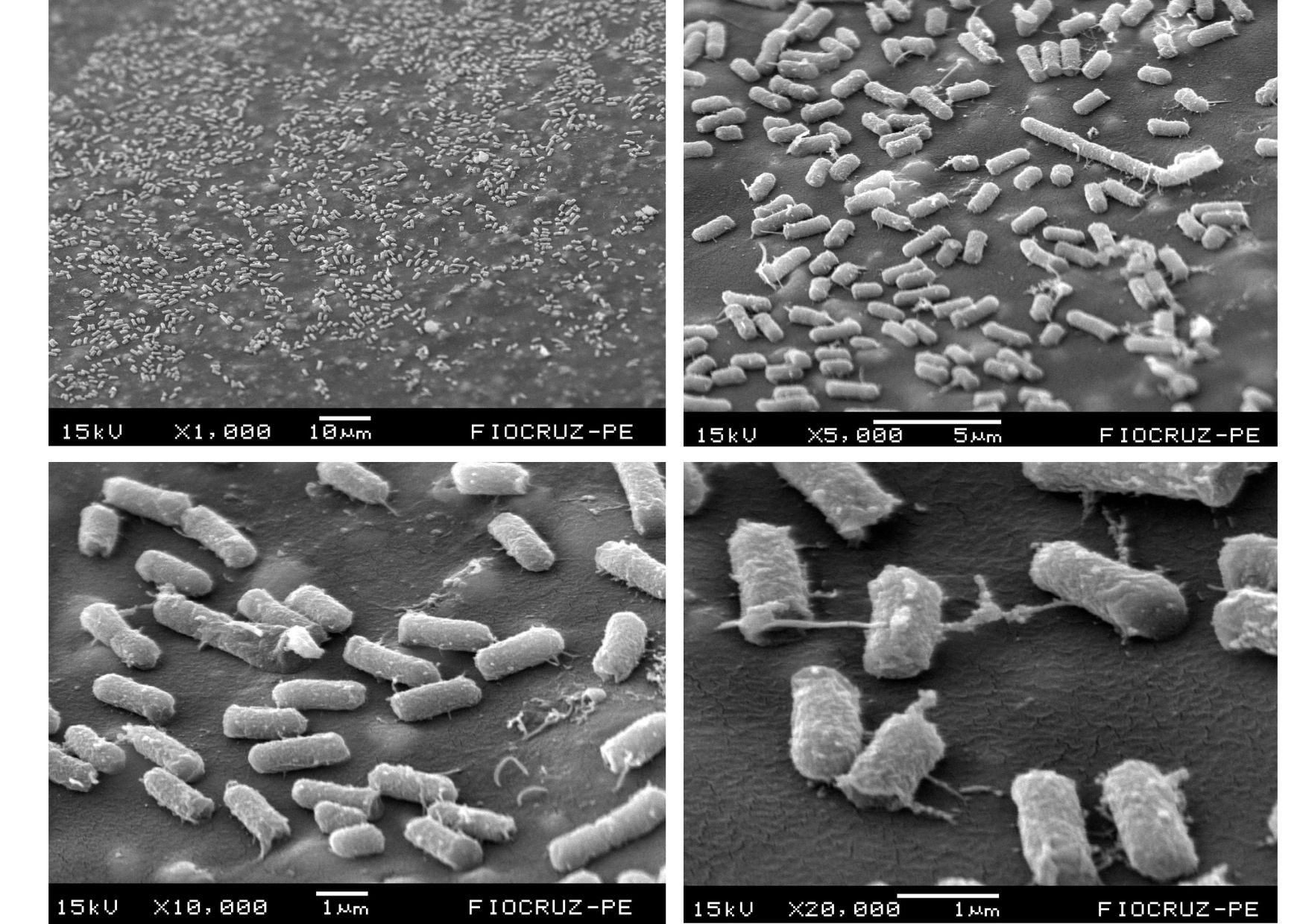


Fig S4. Representative SEM micrographs of 48 h *Escherichia coli* biofilms after sonication treatment at different magnifications (1 000 x, 5 000 x, 10 000 x, 20 000 x).


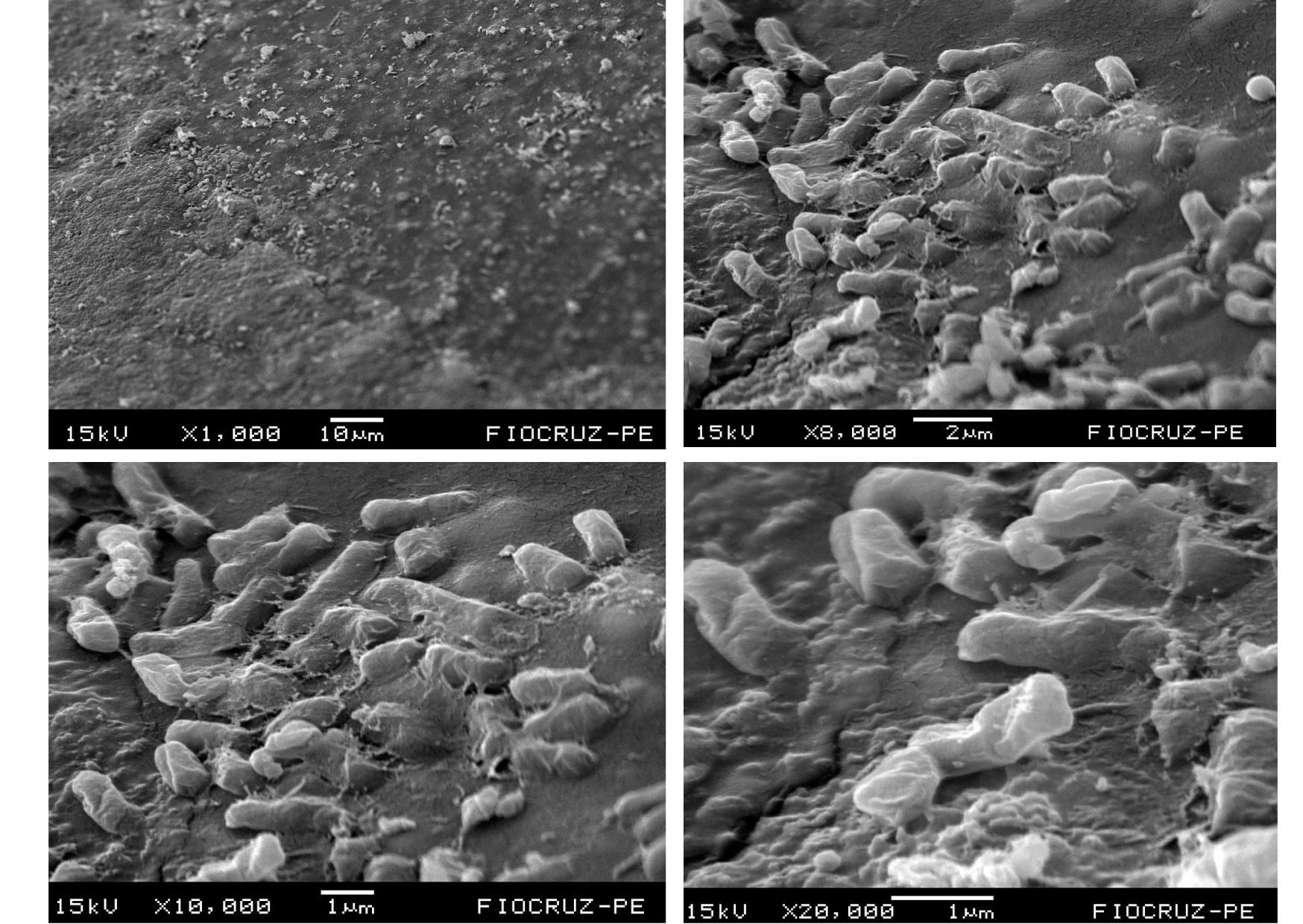


Fig S5. Representative SEM micrographs of 72 h control *Escherichia coli* biofilms at different magnifications (1 000 x, 8 000 x, 10 000 x, 20 000 x).


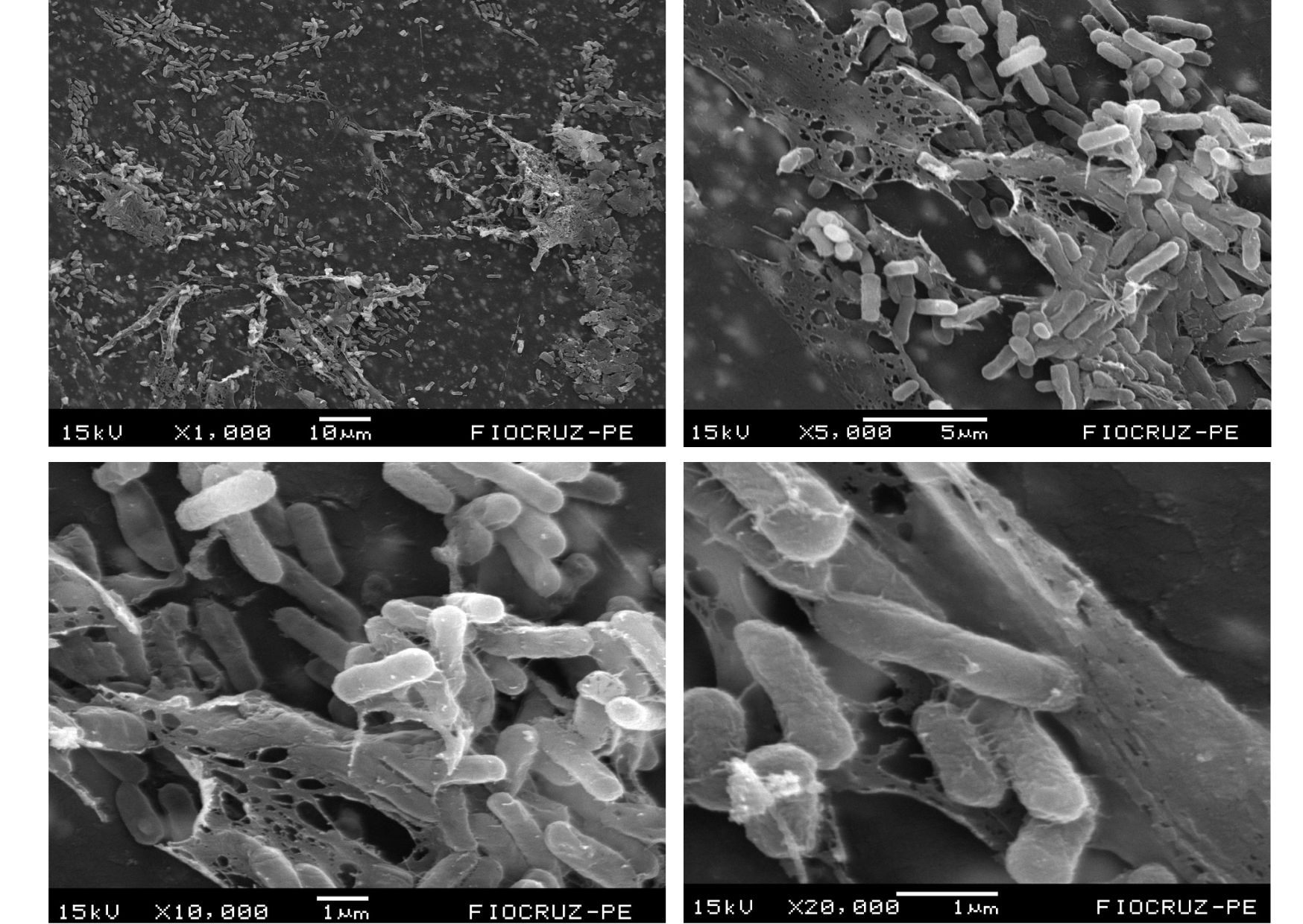


Fig S6. Representative SEM micrographs of 72 h *Escherichia coli* biofilms after sonication treatment at different magnifications (1 000 x, 5 000 x, 10 000 x, 20 000 x).


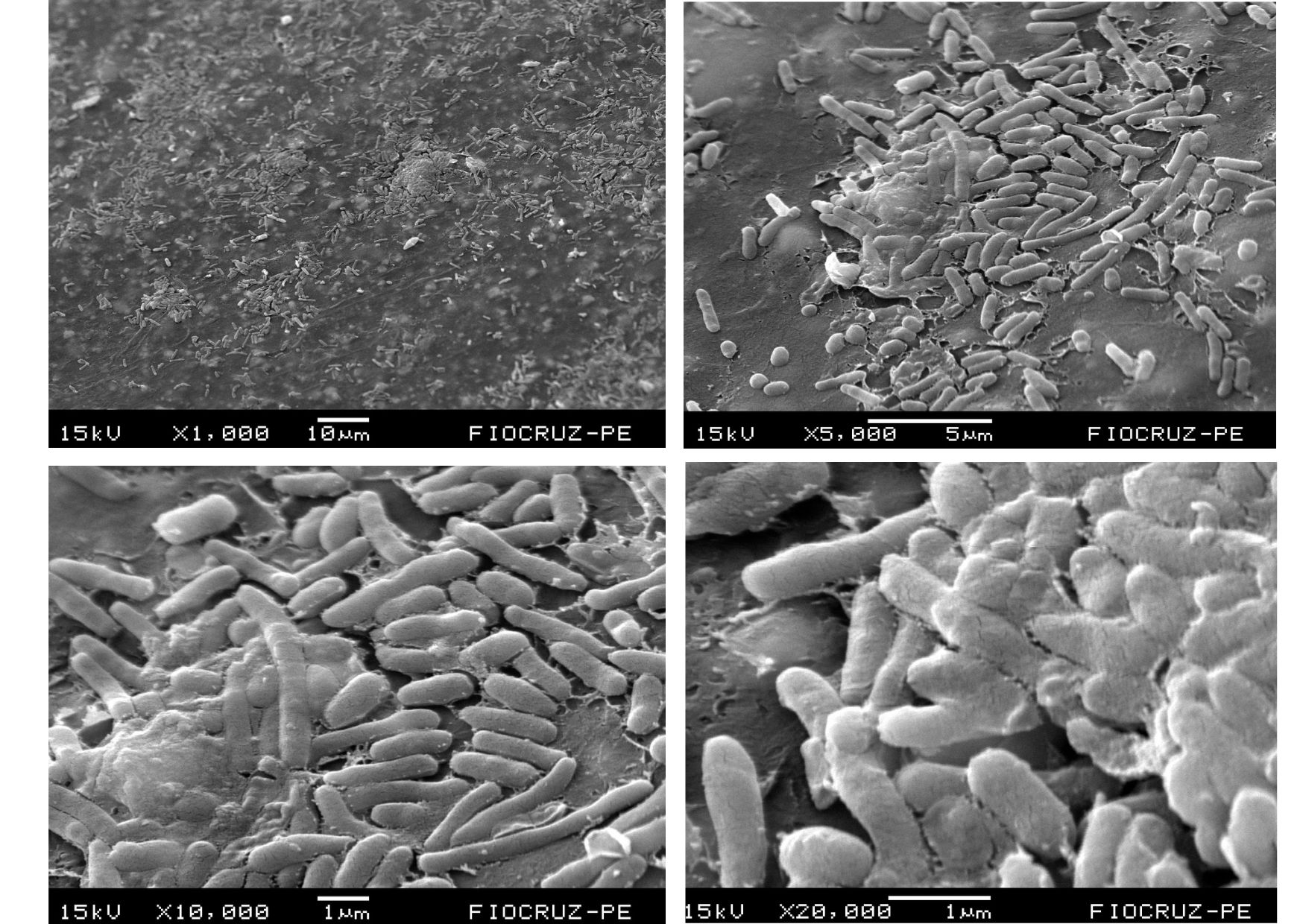


Fig S7. Representative SEM micrographs of 24 h control *Pseudomonas aeruginosa* biofilms at different magnifications (1 000 x, 5 000 x, 10 000 x, 20 000 x).


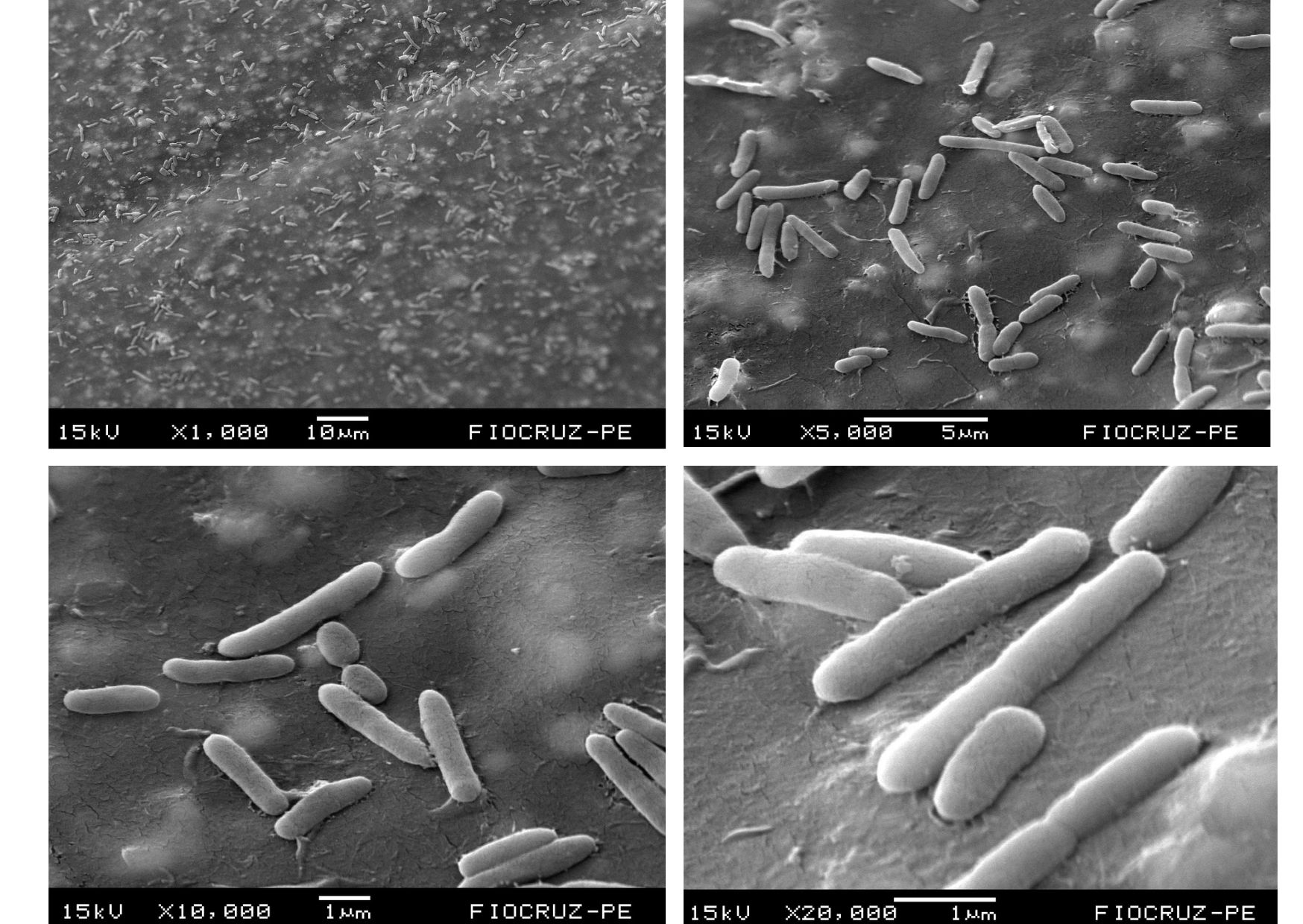


Fig S8. Representative SEM micrographs of 24 h *Pseudomonas aeruginosa* biofilms after sonication treatment at different magnifications (1 000 x, 5 000 x, 10 000 x, 20 000 x).


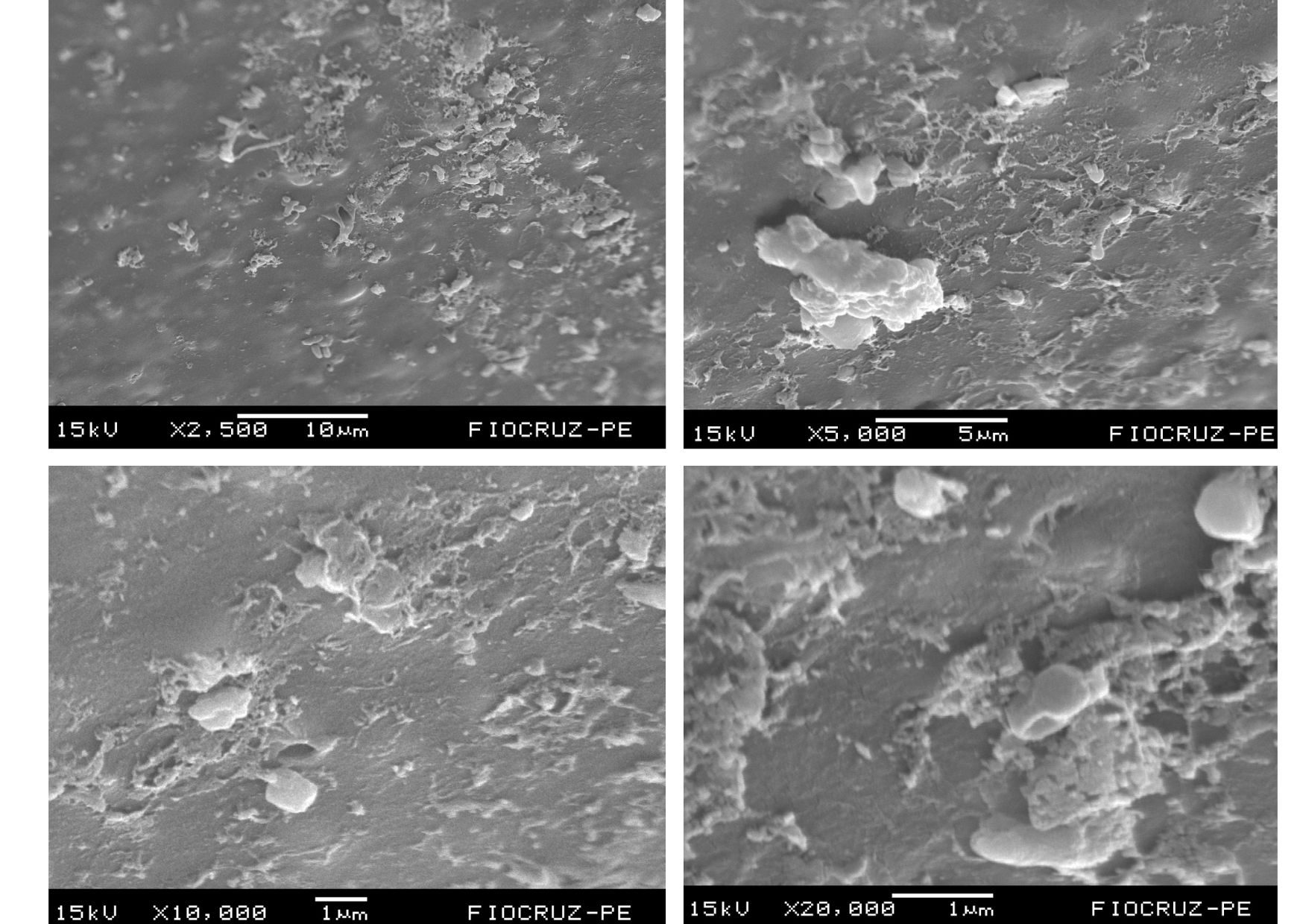


Fig S9. Representative SEM micrographs of 48 h control *Pseudomonas aeruginosa* biofilms at different magnifications (2 500 x, 5 000 x, 10 000 x, 20 000 x).


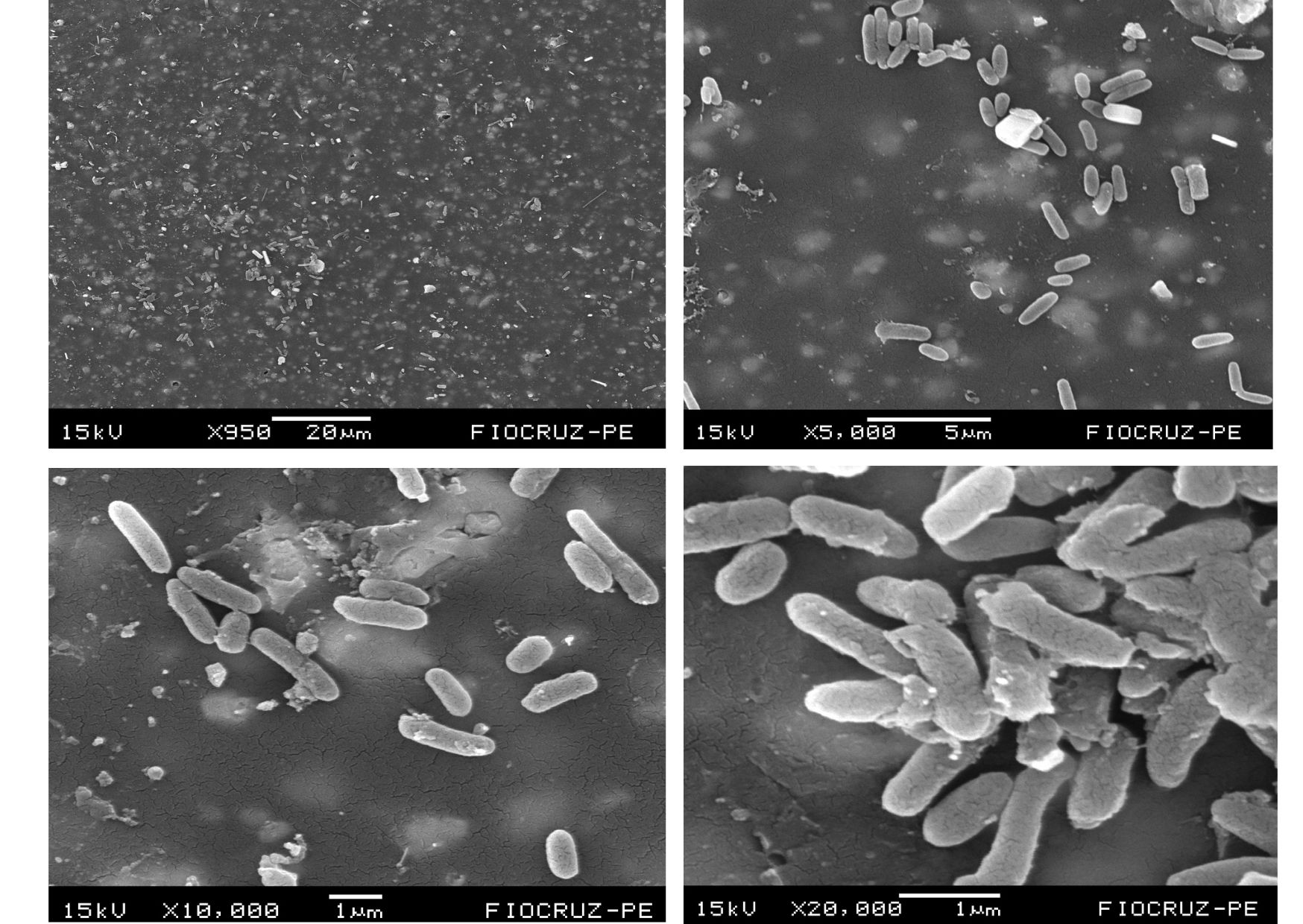


Fig S10. Representative SEM micrographs of 48 h *Pseudomonas aeruginosa* biofilms after sonication treatment at different magnifications (950 x, 5 000 x, 10 000 x, 20 000 x).


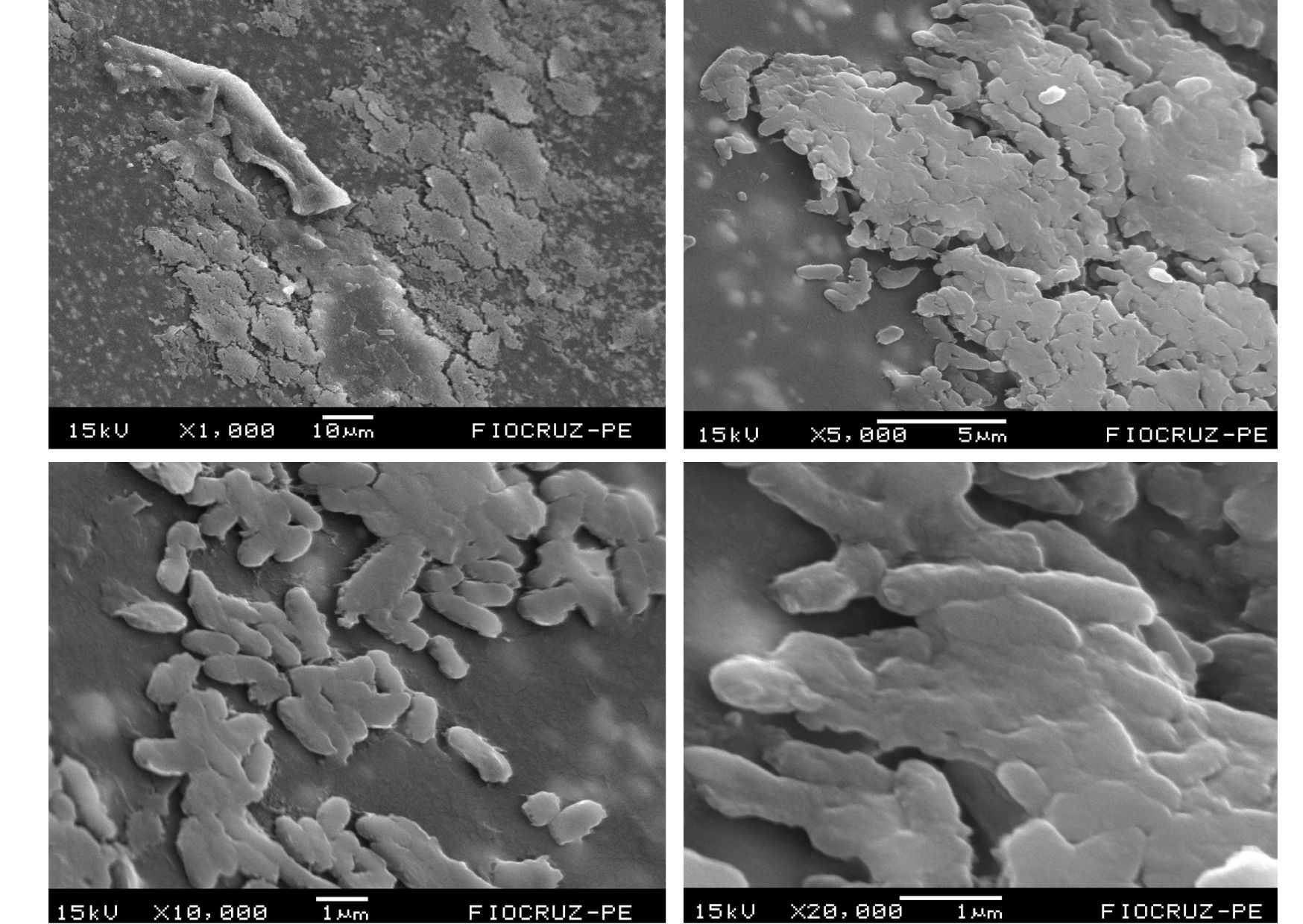


Fig S11. Representative SEM micrographs of 72 h control *Pseudomonas aeruginosa* biofilms at different magnifications (1 000 x, 5 000 x, 10 000 x, 20 000 x).


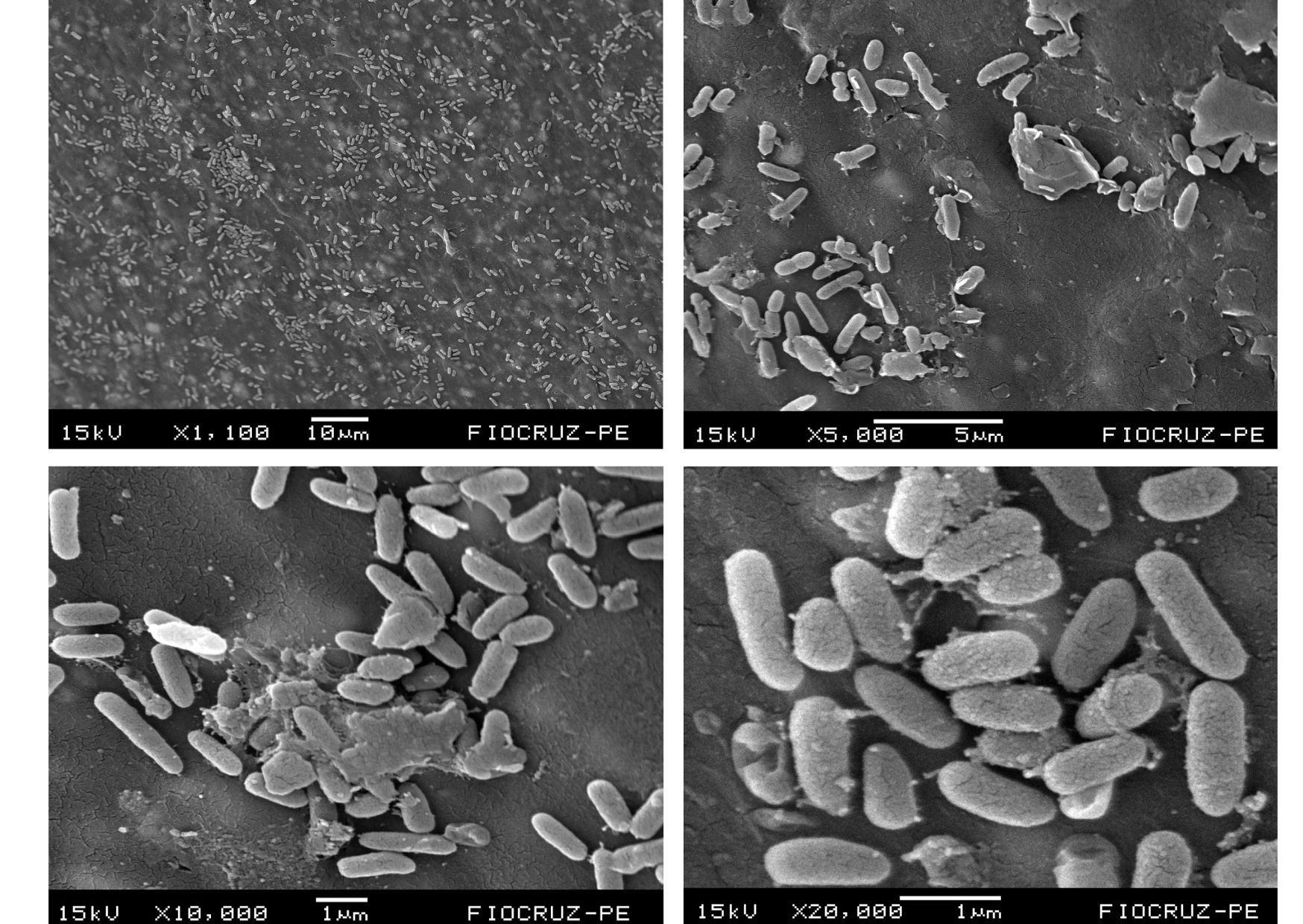


Fig S12. Representative SEM micrographs of 72 h *Pseudomonas aeruginosa* biofilms after sonication treatment at different magnifications (1 100 x, 5 000 x, 10 000 x, 20 000 x).


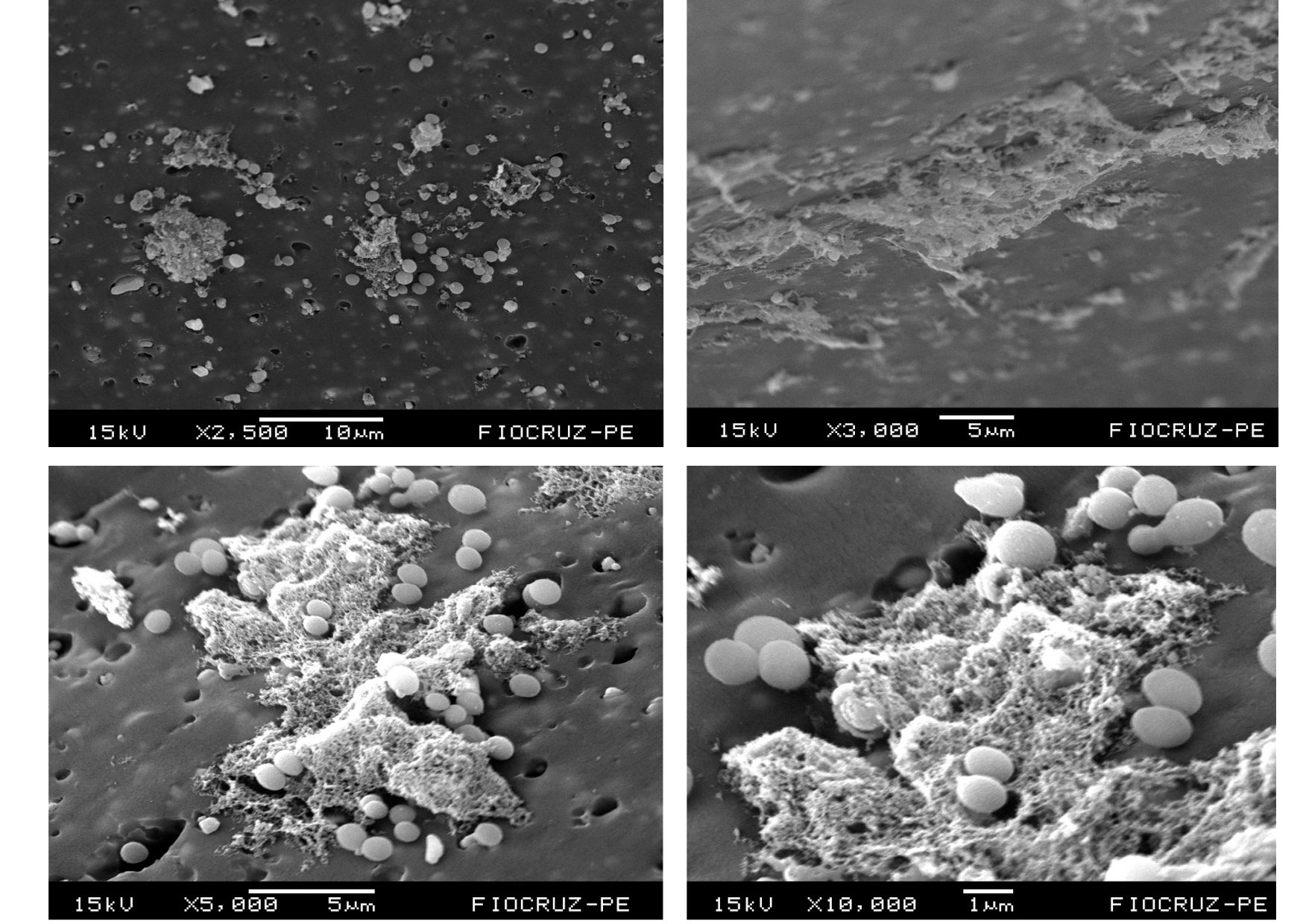


Fig S13. Representative SEM micrographs of 24 h control *Staphylococcus aureus* biofilms at different magnifications (2 500 x, 3 000 x, 5 000 x, 10 000 x).


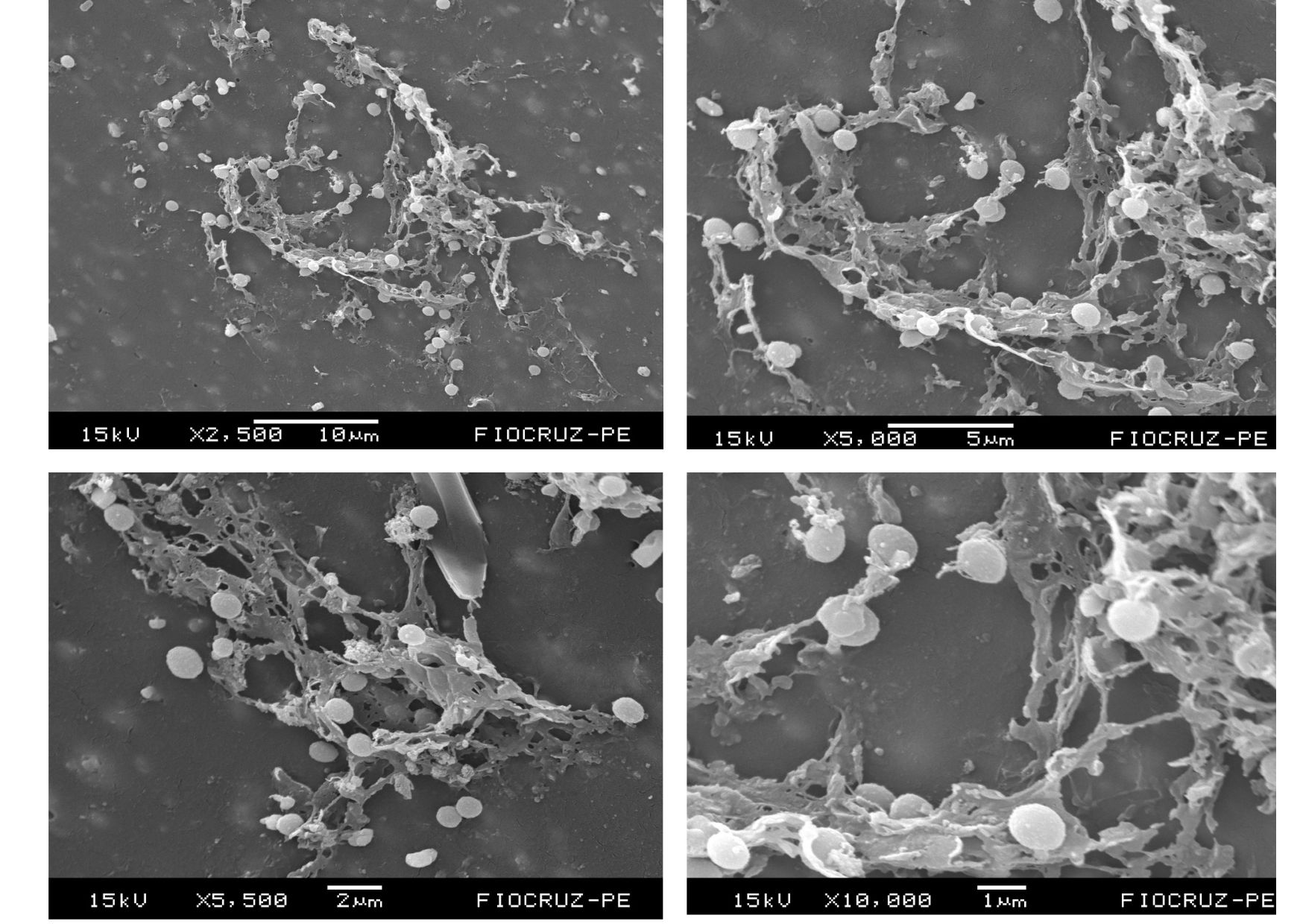


Fig S14. Representative SEM micrographs of 24 h *Staphylococcus aureus* biofilms after sonication treatment at different magnifications (2 500 x, 5 000 x, 5 500 x, 10 000 x).


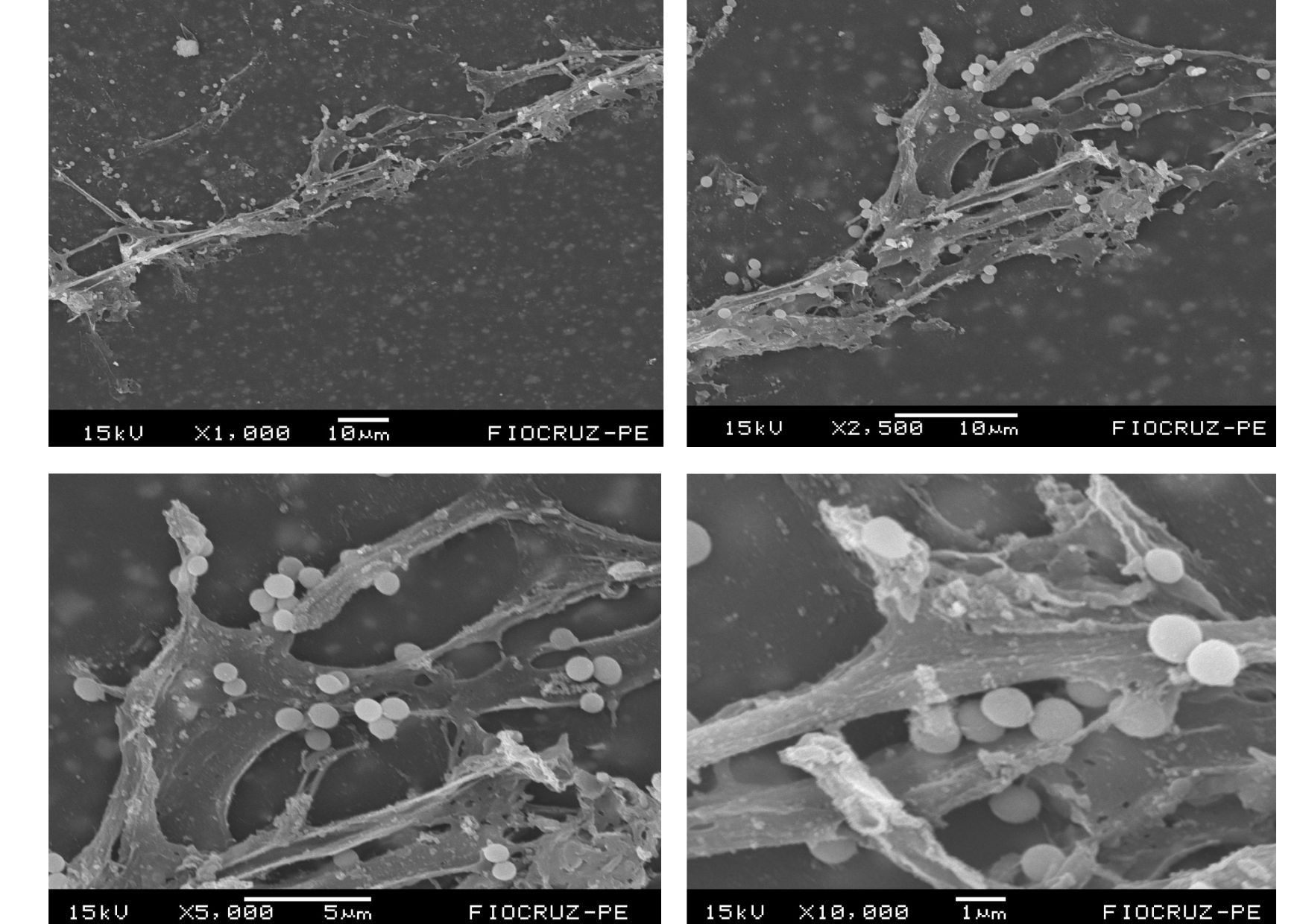


Fig S15. Representative SEM micrographs of 48 h control *Staphylococcus aureus* biofilms at different magnifications (1 000 x, 2 500 x, 5 000 x, 10 000 x).


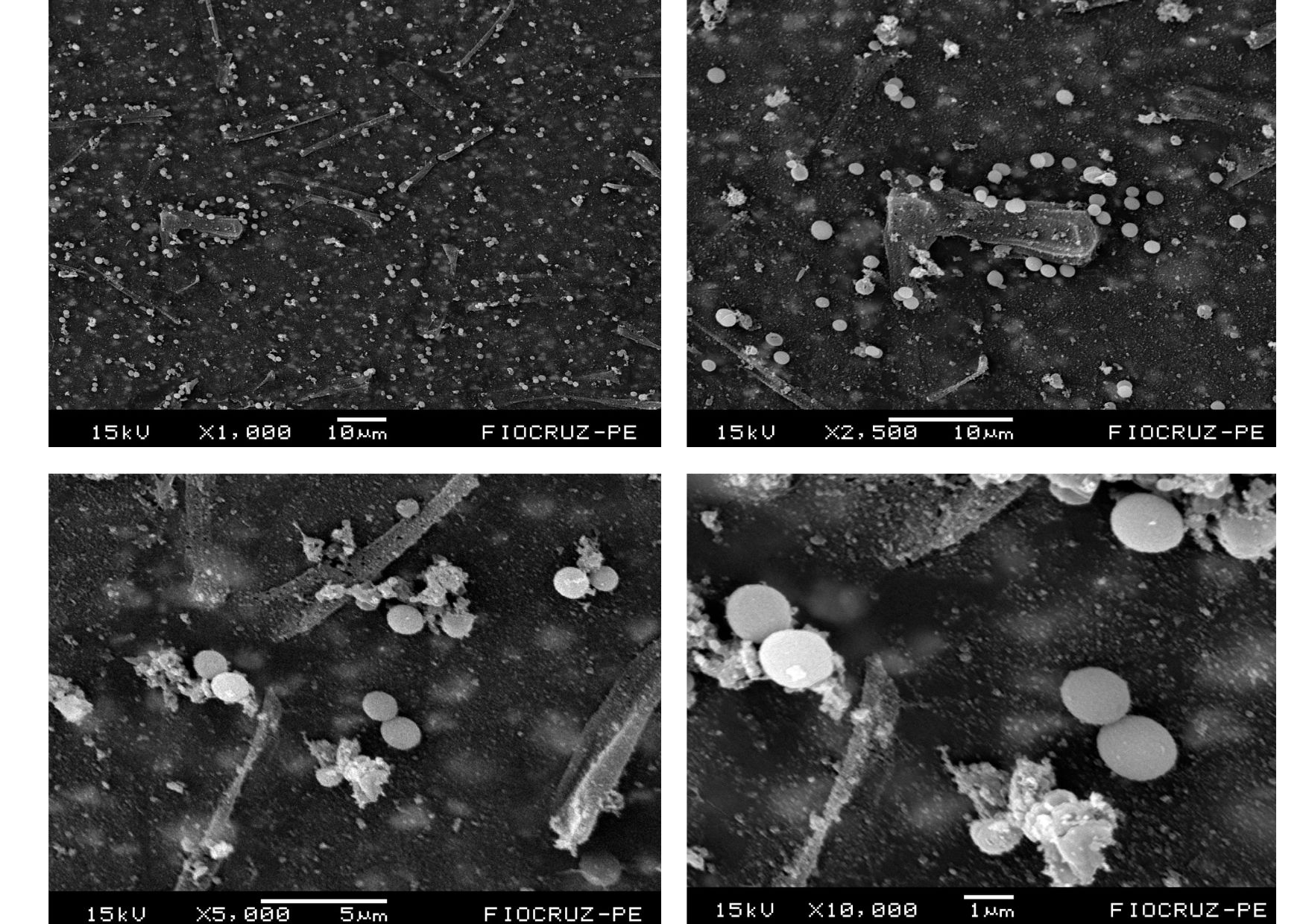


Fig S16. Representative SEM micrographs of 48 h *Staphylococcus aureus* biofilms after sonication treatment at different magnifications (1 000 x, 2 500 x, 5 000 x, 10 000 x).


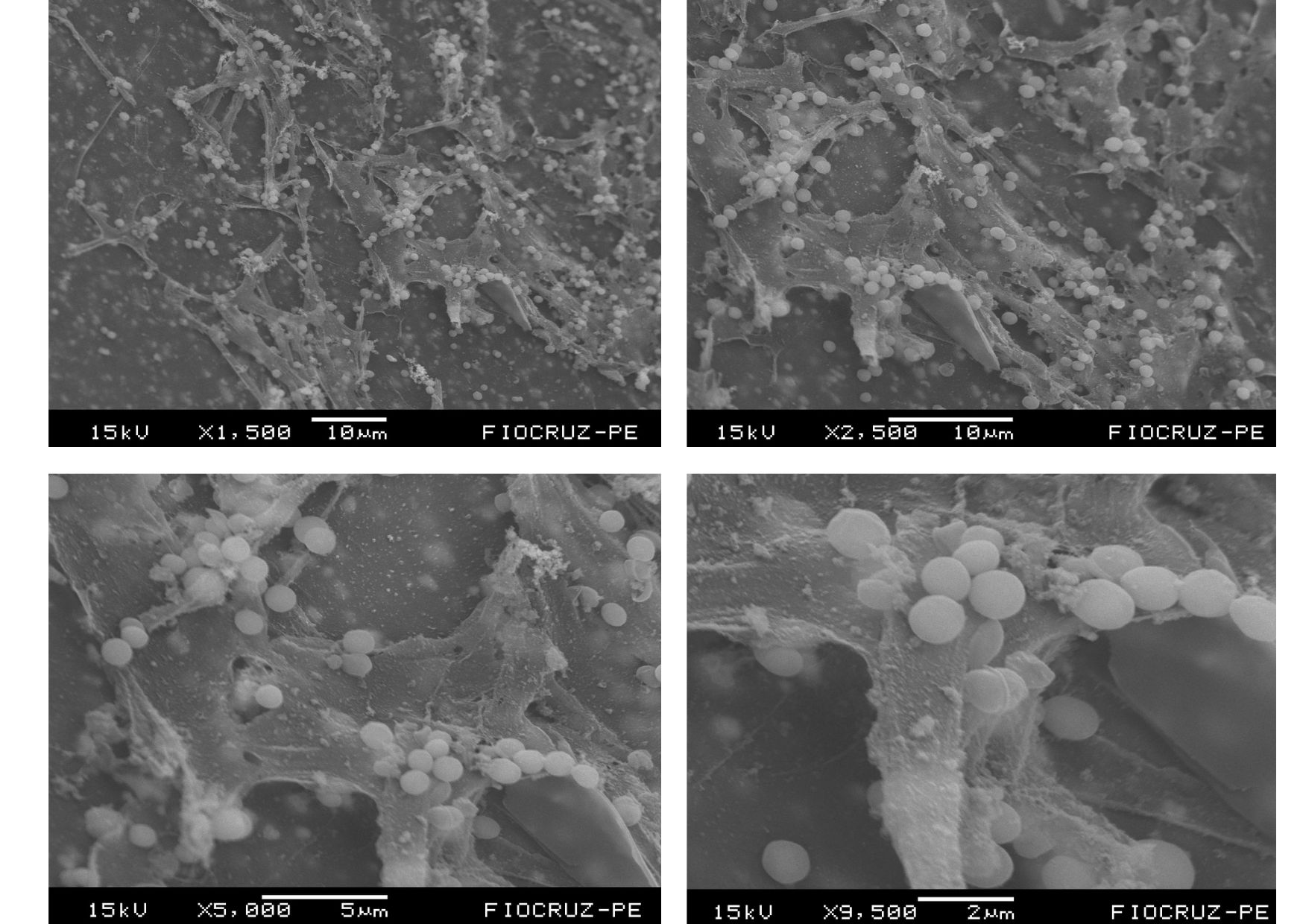


Fig S17. Representative SEM micrographs of 72 h control *Staphylococcus aureus* biofilms at different magnifications (1 500 x, 2 500 x, 5 000 x, 9 500 x).


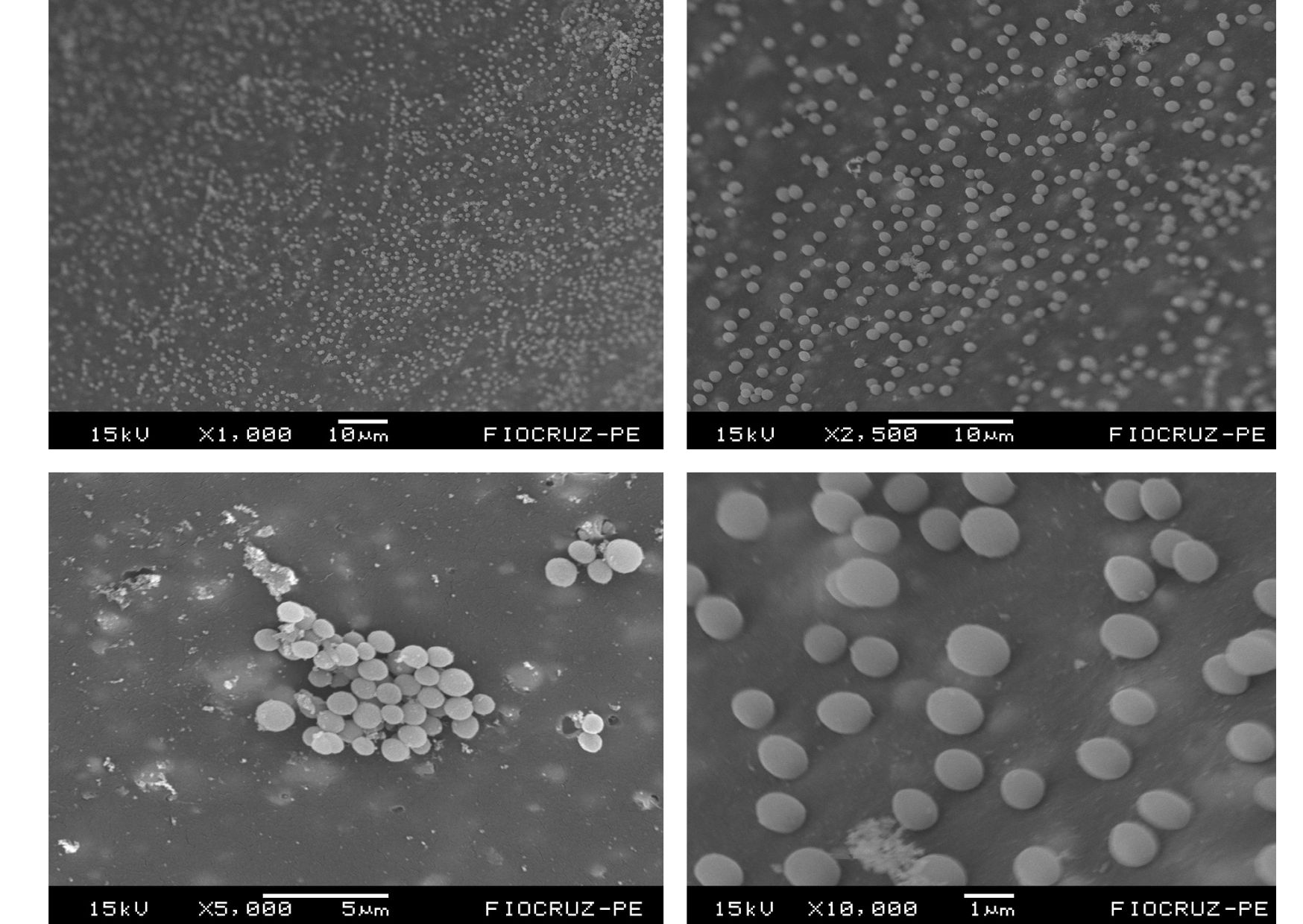


Fig S18. Representative SEM micrographs of 48 h *Staphylococcus aureus* biofilms after sonication treatment at different magnifications (1 000 x, 2 500 x, 5 000 x, 10 000 x).


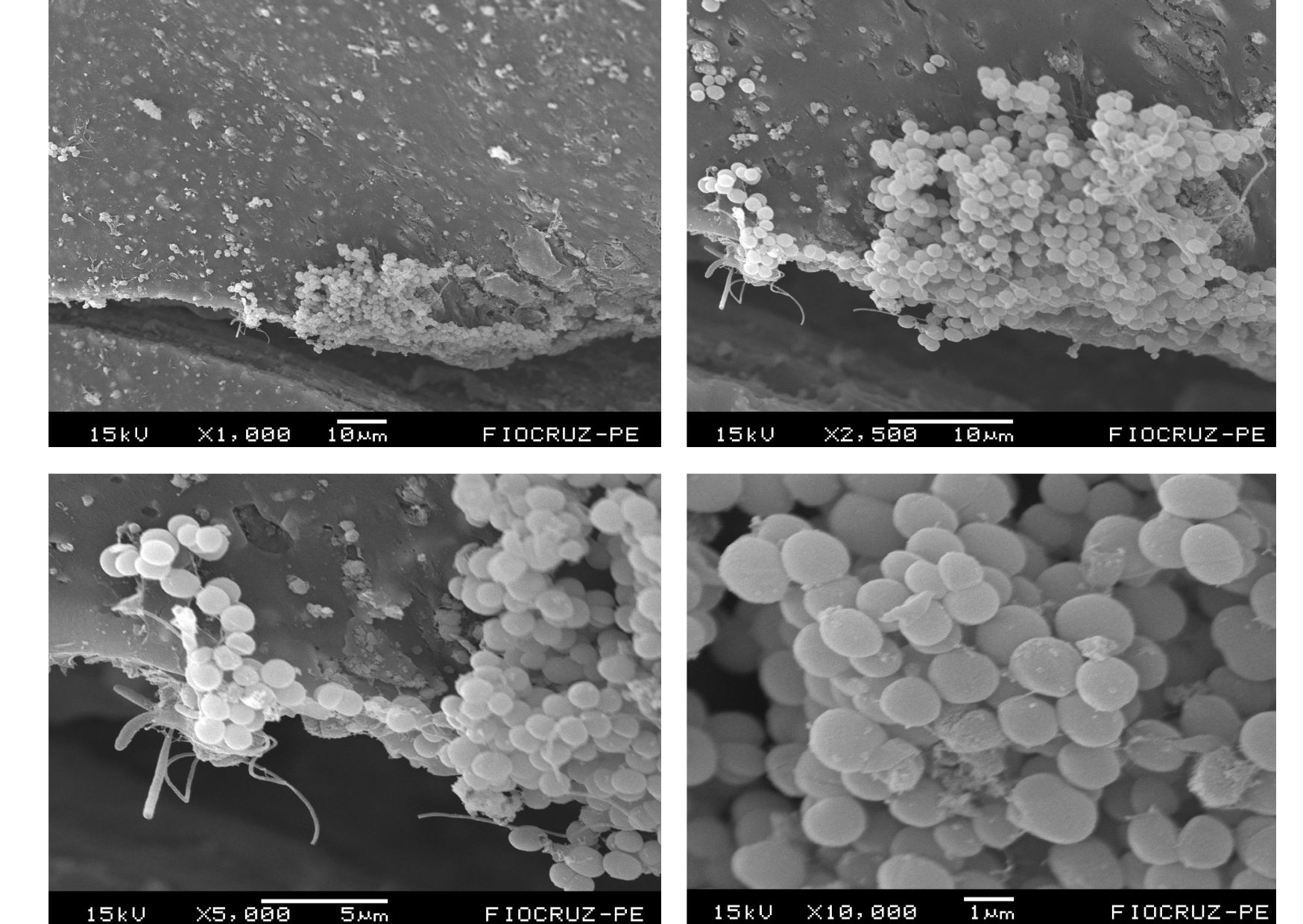


Fig S20. Representative SEM micrographs of 24 h control *Staphylococcus epidermidis* biofilms at different magnifications (1 000 x, 2 500 x, 5 000 x, 10 000 x).


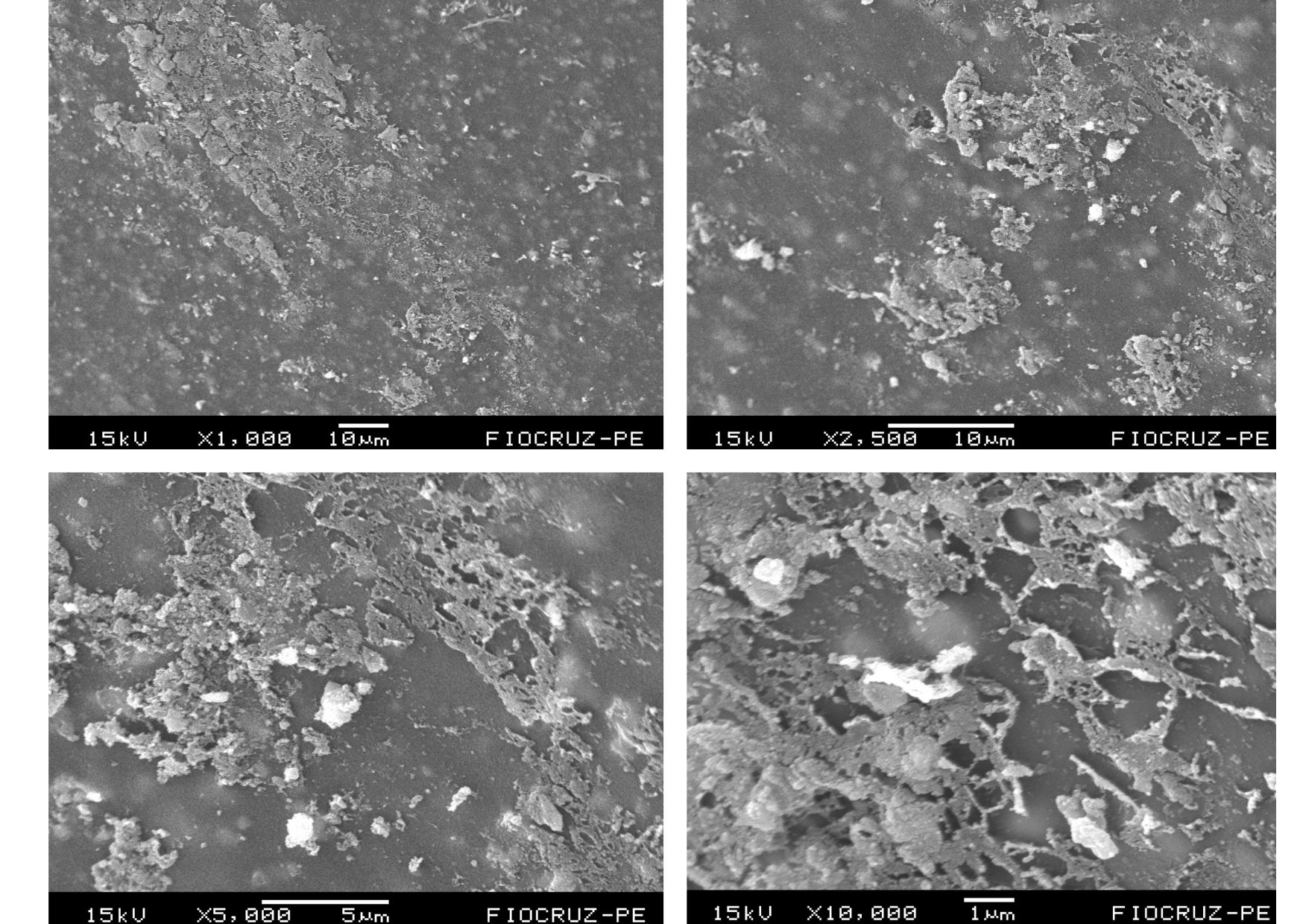


Fig S21. Representative SEM micrographs of 24 h *Staphylococcus epidermidis* biofilms after sonication treatment at different magnifications (1 000 x, 2 500 x, 5 000 x, 10 000 x).


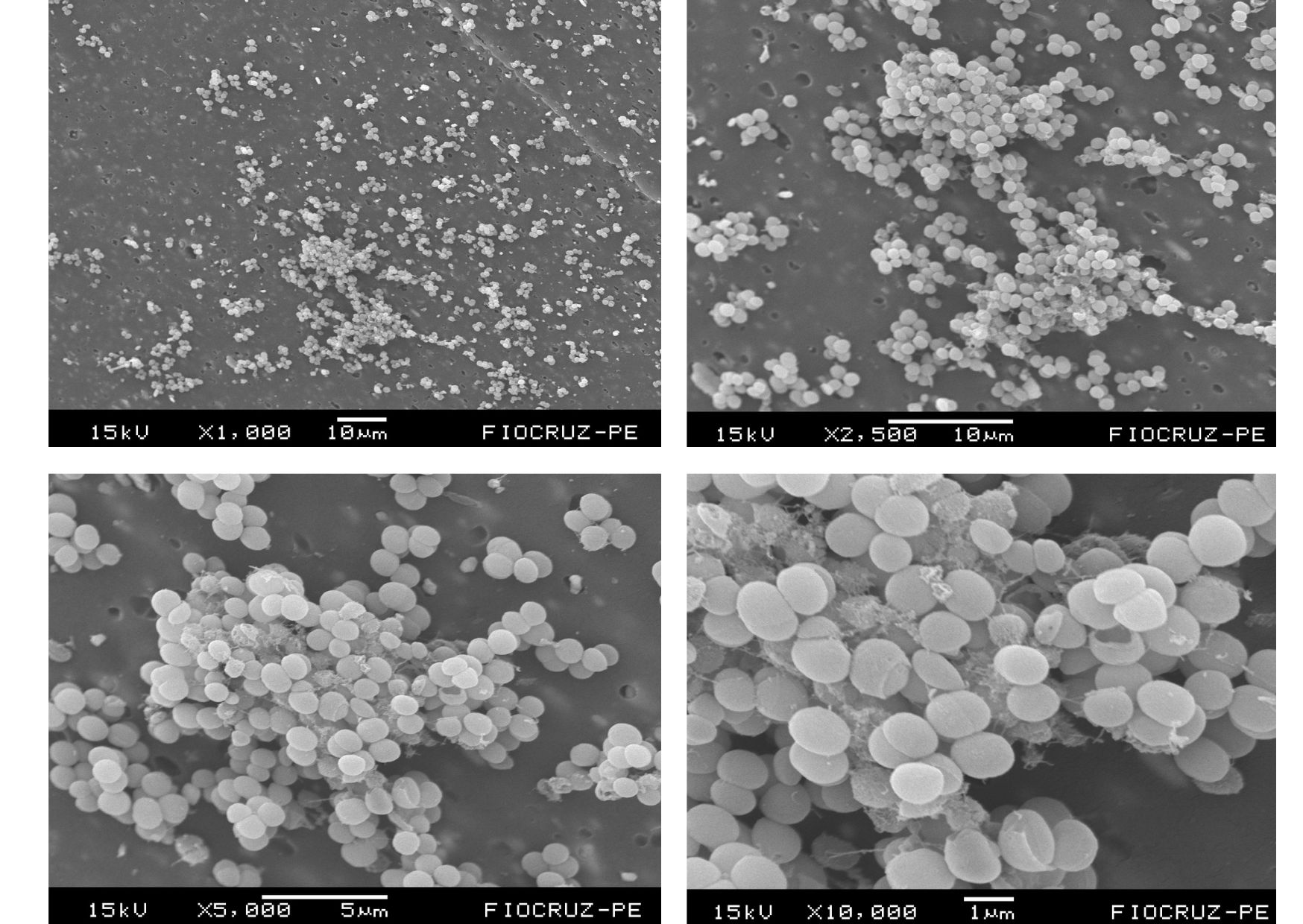


Fig S22. Representative SEM micrographs of 48 h control *Staphylococcus epidermidis* biofilms at different magnifications (1 000 x, 2 500 x, 5 000 x, 10 000 x).


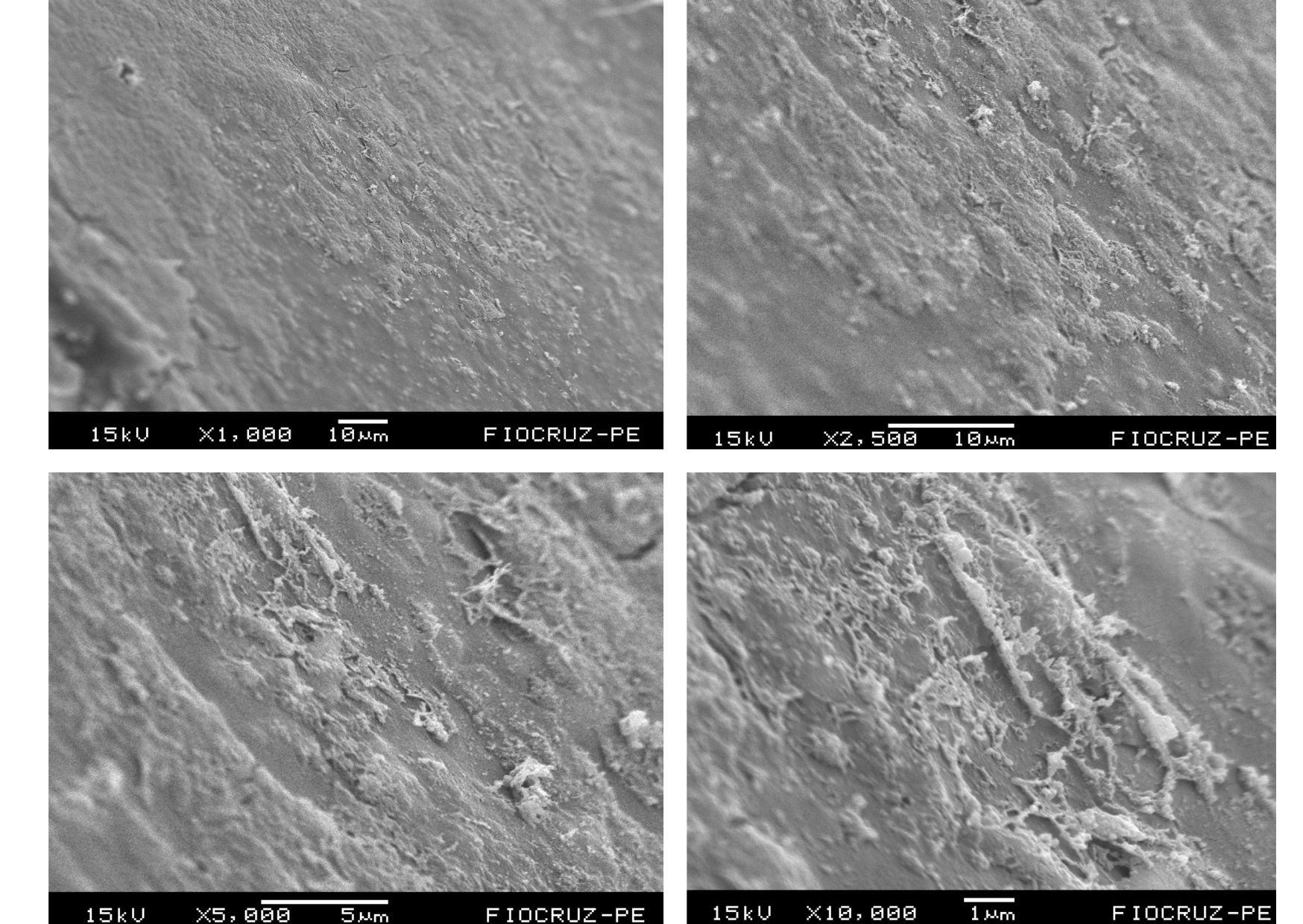


Fig S23. Representative SEM micrographs of 48 h *Staphylococcus epidermidis* biofilms after sonication treatment at different magnifications (1 000 x, 2 500 x, 5 000 x, 10 000 x).


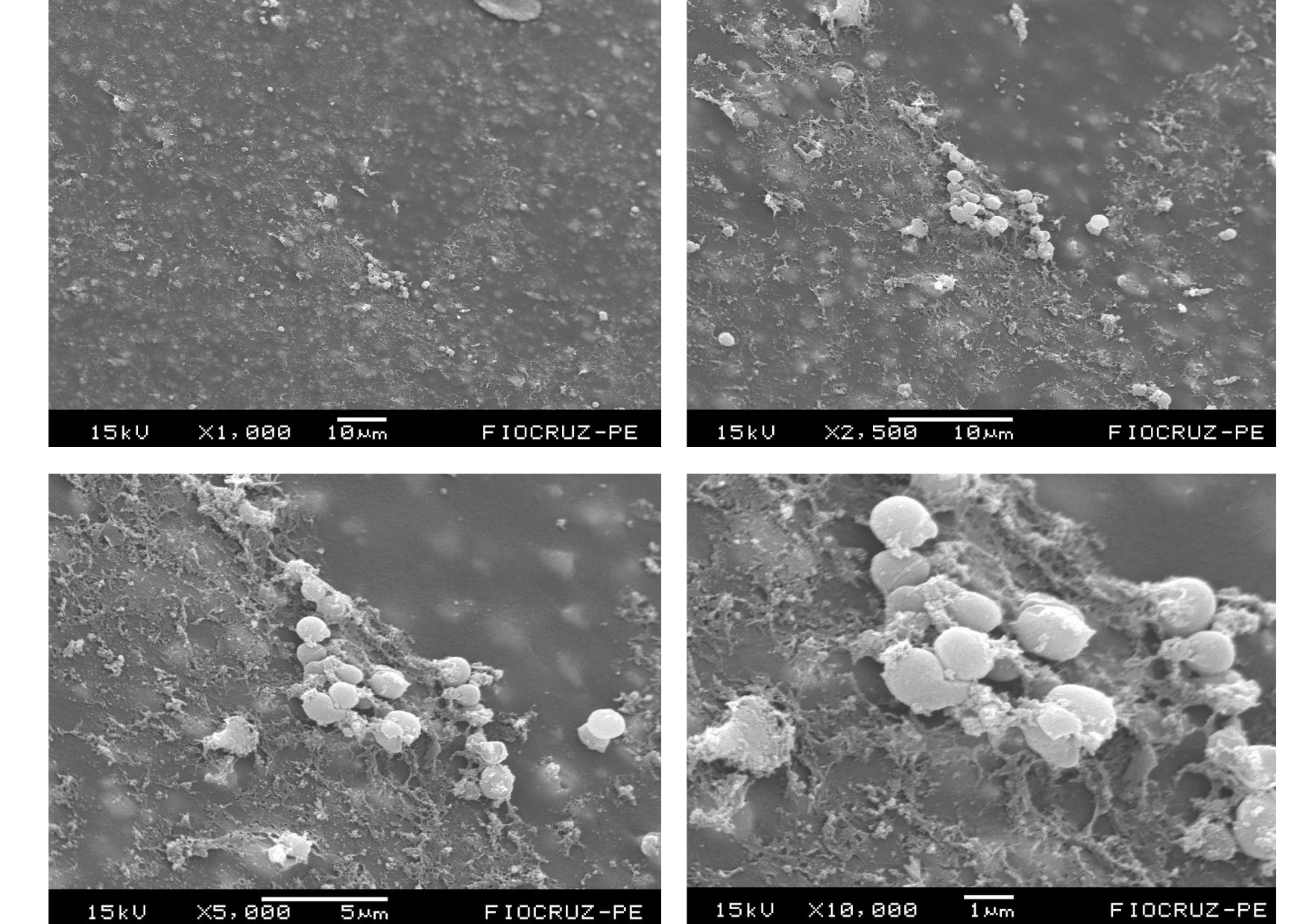


Fig S24. Representative SEM micrographs of 72 h control *Staphylococcus epidermidis* biofilms at different magnifications (1 000 x, 2 500 x, 5 000 x, 10 000 x).


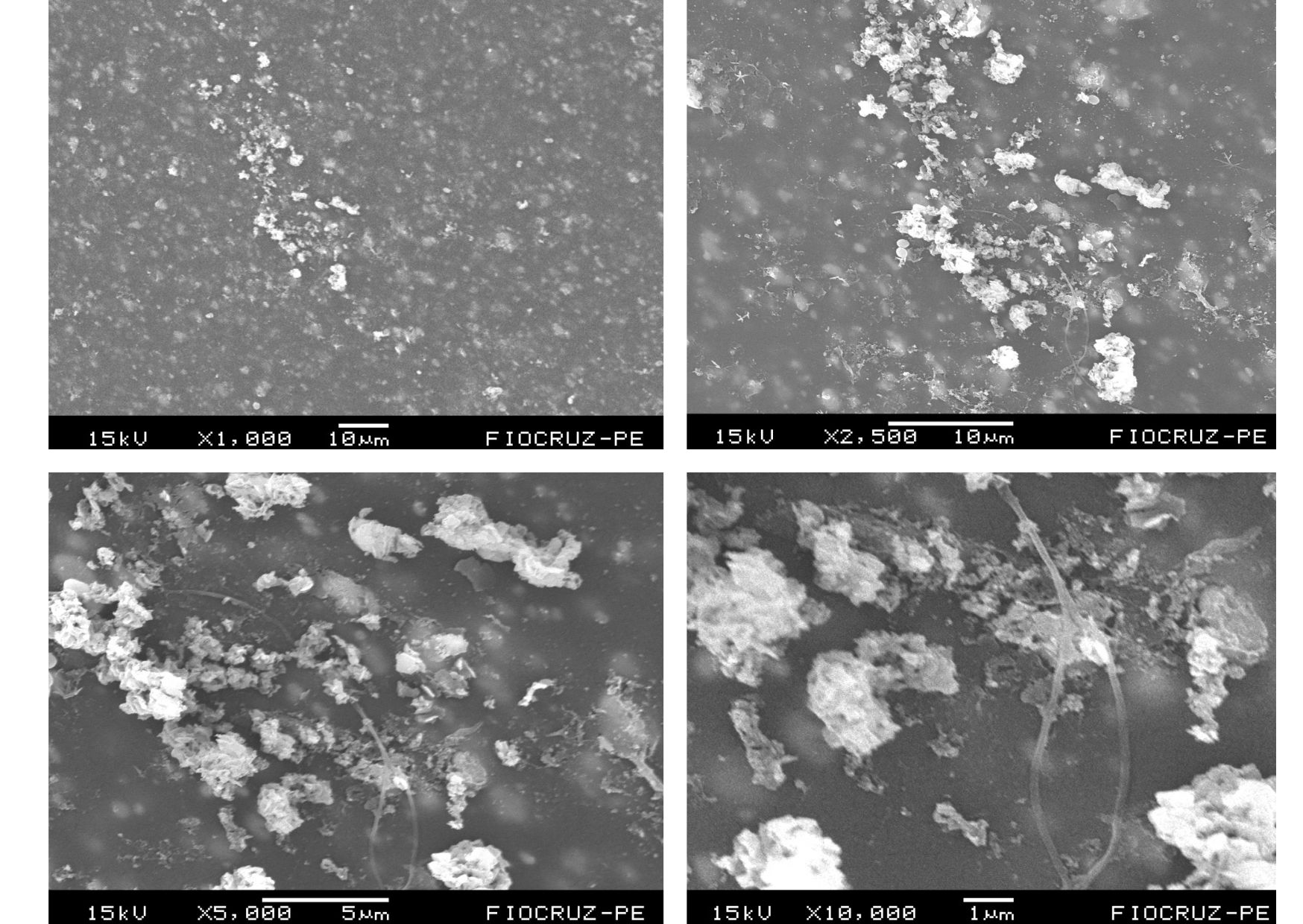


Fig S26. Representative SEM micrographs of 72 h *Staphylococcus epidermidis* biofilms after sonication treatment at different magnifications (1 000 x, 2 500 x, 5 000 x, 10 000 x).


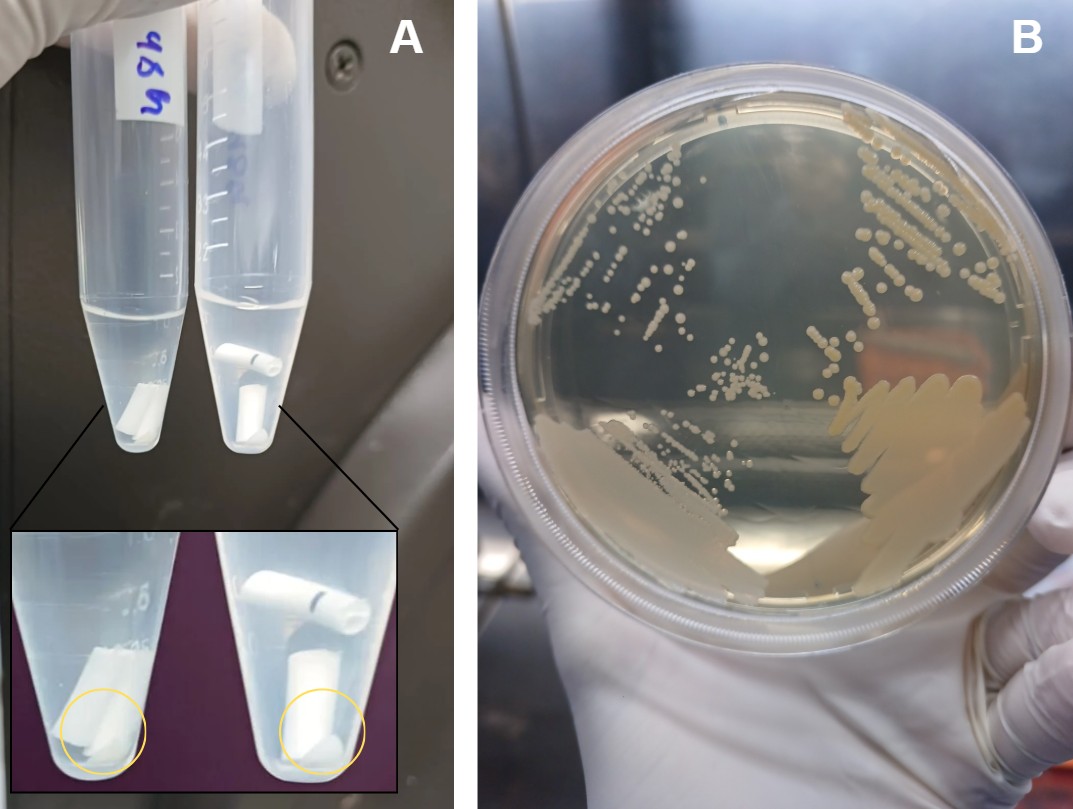


Fig S27. Recovery of culturable bacteria after sonication. (A) Pellet formed after centrifugation of the sonication fluid collected from the catheter segments, indicating the release of microbial material. (B) Growth of bacterial colonies on BHI agar after 18–24 h of incubation at 37 °C, demonstrating the culturability of cells detached by sonication. A volume of 10 µL of the sonication fluid was plated, and the agar plate was divided into four quadrants, with two quadrants corresponding to each bacterial strain inoculated from the same sonicated suspension. This layout allows a qualitative comparison of bacterial recovery between the two Gram-negative species following sonication.

| **Species** | **Condition** | **Image** | **No. of particles / cells** | **Mean area of biofilm structures (µm²)** | **Cell density (cells/µm²)** |
| --- | --- | --- | --- | --- | --- |
| *S. aureus* | Sonicated | Img1 | 14 | 0.241 | — |
| *S. aureus* | Sonicated | Img2 | 15 | 0.227 | — |
| *S. aureus* | Sonicated | Img3 | 7 | 0.531 | — |
| **S. aureus** | **Sonicated (mean ± SD)** | — | **12.0 ± 4.4** | **0.333 ± 0.172** | — |
| *S. aureus* | Control | Img1 | 6 | 0.864 | — |
| *S. aureus* | Control | Img2 | 3 | 0.197 | — |
| *S. aureus* | Control | Img3 | 7 | 0.136 | — |
| **S. aureus** | **Control (mean ± SD)** | — | **5.3 ± 2.1** | **0.399 ± 0.397** | — |
| *S. epidermidis* | Control | Img1 | 4 | 0.363 | — |
| *S. epidermidis* | Control | Img2 | 7 | 0.176 | — |
| *S. epidermidis* | Control | Img3 | 8 | 0.173 | — |
| ***S. epidermidis*** | **Control (mean ± SD)** | — | **6.3 ± 2.1** | **0.237 ± 0.108** | — |
| *S. epidermidis* | Sonicated | Img1 | 7 | 0.167 | — |
| *S. epidermidis* | Sonicated | Img2 | 3 | 0.125 | — |
| *S. epidermidis* | Sonicated | Img3 | 3 | 0.171 | — |
| ***S. epidermidis*** | **Sonicated (mean ± SD)** | — | **4.3 ± 2.3** | **0.154 ± 0.025** | — |
| *E. coli* | Control | Img1 | 11 | — | 0.0281 |
| *E. coli* | Control | Img2 | 15 | — | 0.0300 |
| *E. coli* | Control | Img3 | 22 | — | 0.0448 |
| ***E. coli*** | **Control (mean ± SD)** | — | **16.0 ± 5.6** | — | **0.0343 ± 0.0093** |
| *E. coli* | Sonicated | Img1 | 34 | — | 0.0692 |
| *E. coli* | Sonicated | Img2 | 43 | — | 0.0875 |
| *E. coli* | Sonicated | Img3 | 31 | — | 0.0611 |
| ***E. coli*** | **Sonicated (mean ± SD)** | — | **36.0 ± 6.2** | — | **0.0726 ± 0.0136** |
| *P. aeruginosa* | Control | Img1 | 17 | — | 0.0357 |
| *P. aeruginosa* | Control | Img2 | 44 | — | 0.0953 |
| *P. aeruginosa* | Control | Img3 | 10 | — | 0.0210 |
| ***P. aeruginosa*** | **Control (mean ± SD)** | — | **23.7 ± 18.3** | — | **0.0507 ± 0.0390** |
| *P. aeruginosa* | Sonicated | Img1 | 103 | — | 0.2129 |
| *P. aeruginosa* | Sonicated | Img2 | 69 | — | 0.1449 |
| *P. aeruginosa* | Sonicated | Img3 | 50 | — | 0.1050 |
| **P. aeruginosa** | **Sonicated (mean ± SD)** | — | **74.0 ± 26.6** | — | **0.1543 ± 0.0549** |

Table S1. Raw data from image-based quantification of biofilm structures and cellular dispersion with and without sonication.
